# Intercommunity interactions and killings in central chimpanzees (*Pan troglodytes troglodytes*) from Loango National Park, Gabon

**DOI:** 10.1007/s10329-021-00921-x

**Published:** 2021-06-17

**Authors:** Laura Martínez-Íñigo, Pauline Baas, Harmonie Klein, Simone Pika, Tobias Deschner

**Affiliations:** 1 Interim group Primatology. Max Planck Institute for Evolutionary Anthropology, Deutscher Platz 6, 04103 Leipzig, Germany; 2 Osnabrück University, Institute of Cognitive Science, Comparative BioCognition, Artilleriestrasse 34, 49076 Osnabrück, Germany

**Keywords:** Chimpanzees, between-group competition, Imbalance-of-power, Temporal landscape partitioning, Home range overlap, Infanticide, Camera traps

## Abstract

Intercommunity competition in chimpanzees (*Pan troglodytes*) has been widely studied in eastern (*P. t. schweinfurthii*) and western (*P. t. verus*) communities. Both subspecies show hostility towards neighboring communities but differ in rates of lethal attacks and female involvement. However, relatively little is known about the territorial behavior of the two other subspecies, central (*P. t. troglodytes*) and Nigeria-Cameroon chimpanzees (*P. t. ellioti*). Here, we present the first insights into intercommunity interactions of individuals of a community of central chimpanzees living in the Loango National Park in Gabon. The presence of individuals of neighboring communities in the Rekambo home range was assessed using 27 camera traps. Information was compiled on intergroup interactions recorded before (2005–2016) and after (January 2017–June 2019) the habituation of the community. Individuals from neighboring communities entered the core area, where nine out of 16 recorded intercommunity encounters occurred. Males were the main participants in territorial patrols and intercommunity aggressions. Females were part of all six territorial patrols recorded and dependent offspring participated in five patrols. Females were involved in intercommunity aggression in five out of twelve recorded encounters in which there was visual contact between communities. While the intercommunity encounter rate was lower than that reported across most other long-term chimpanzee sites, the annual intercommunity killing rate was among the highest. These results suggest that the frequency of lethal attacks at Loango is comparable to that reported for the eastern subspecies. In contrast, female involvement in intercommunity interactions mirrors that of the western subspecies.

**Supplementary Information:**

The online version contains supplementary material available at 10.1007/s10329-021-00921-x.

## Introduction

Social animals live in groups usually surrounded by other groups of conspecifics that compete against each other for limited resources (Tanner 2006; Cassidy et al. 2017; Strong et al. 2018). Participation in between-group conflicts varies with individual fitness payoffs, which relate to individual features such as dispersal status and sex (Kitchen and Beehner 2007). Multi-male-multi-female groups are often unsuccessful at excluding other groups from their home ranges (Willems et al. 2013). However, this pattern differs in cases where the larger sex stays in their natal group. This is the case of chimpanzees (*Pan troglodytes*) (Nishida 1979; Goodall 1986; Boesch and Boesch-Achermann 2000; Willems et al. 2013).

Chimpanzees live in fission–fusion communities of up to 200 individuals with multiple adult females and males and their offspring, but spend most of their time in smaller parties of varying size and composition (e.g., Nishida 1979; Goodall 1986; Boesch and Boesch-Achermann 2000; Watts and Mitani 2015). Females usually transfer between communities after reaching sexual maturity, while males, larger than females, stay in their natal group. Intercommunity interactions are infrequent and hostile, and the main participants are males (see Furuichi 2020 for a review). Overlapping areas between communities can reach up to 30–50% of their territories (Nishida 1979; Goodall 1986), while exclusive core areas may be less than 23% of the total home range size (Herbinger et al. 2001; Amsler 2009). Parties patrol territorial boundaries, sometimes intruding deep (> 1 km; deep incursion) into the territory of other communities (Nishida et al. 1985; Goodall 1986; Boesch and Boesch-Achermann 2000; Wilson et al. 2004; Mitani and Watts 2005). Patrolling parties respond aggressively (e.g., by charging, chasing, use of threatening displays, contact aggression) when encountering non-community members (Nishida et al. 1985; Goodall 1986; Boesch and Boesch-Achermann 2000; Wilson et al. 2004; Mitani and Watts 2005). Most intercommunity interactions consist of acoustic contacts at long distances (i.e., pant-hoots and drumming, Boesch and Boesch-Achermann 2000; Crofoot and Wrangham 2010) but can escalate into lethal attacks (e.g., Wilson et al. 2014).

Most of our current knowledge of intergroup interactions in chimpanzees stems from studies on several eastern communities (*P. t. schweinfurthii*) (see Furuichi 2020 for a recent review). Males almost exclusively carry out the territorial activities in this subspecies, and killings are relatively frequent (Wilson et al. 2014; Furuichi 2020). Such patterns led to the hypothesis that resident males form coalitions to compete intensely against males from other groups to increase the size of their territory (Williams et al. 2004; Mitani et al. 2010). A larger territory may attract more females into the community and improve the reproduction of intracommunity females (Williams et al. 2004; Mitani et al. 2010). This hypothesis views female chimpanzees as passive entities in intercommunity conflicts. However, studies on communities of western chimpanzees (*P. t. verus*) in the Taï National Park, Côte d'Ivoire, showed a different pattern. For instance, in this subspecies females are frequently involved in territorial activities and contribute significantly to displace individuals of other communities during encounters (Boesch and Boesch-Achermann 2000; Boesch et al. 2008; Wilson et al. 2014; Hashimoto et al. 2020; Lemoine et al. 2020). In addition, killings are less frequent than in eastern chimpanzees (Wilson et al. 2014). In contrast, virtually nothing is known about intercommunity interactions of the other two subspecies, central (*P. t. troglodytes*) and Nigeria-Cameroon (*P. t. ellioti*) chimpanzees (Furuichi 2020).

Hence, the present study aimed to improve our knowledge of chimpanzee territorial behavior by providing insights into the intercommunity relationships of a community of central chimpanzees living in the Loango National Park, Gabon. We used data combining direct observations and camera traps to describe the interactions between the studied community and its neighbors. The analyses focused on intergroup encounter patterns and their location within the territory as well as female and male participation in territorial activities. We compare our results to data from other long-term chimpanzee sites and pinpoint differences and similarities.

## Methods

### Study site and community

The study site was established in 2005 in the Loango National Park, Gabon (2° 04′ S, 9° 33′ E, Boesch et al. 2007). The ecological parameters of the field site are described in detail elsewhere (Boesch et al. 2007; Head et al. 2011).

Based on data from 27 camera traps and direct observations, the Rekambo community consisted of 45 individuals at the end of the data collection period of this study (June 2019). These individuals were eight adult males, 16 adult females, three adolescent males, six adolescent females, and 12 juveniles and infants (following the classification of van Lawick-Goodall 1968). The habituation to human presence was successful for all adult and adolescent males of the community. Furthermore, nine adult females with six infants, five adolescent females, and three juvenile males were regularly observed. The remaining seven adult females and their offspring were mainly seen in association with habituated individuals on camera trap footage. Monthly community size and composition can be found in the electronic supplementary material (ESM) of Martínez-íñigo et al. (in review). Previous genetic and camera trap studies (Arandjelovic et al. 2011; Head et al. 2013) suggested that the Rekambo community is surrounded by a minimum of three other unhabituated communities in the north, the east, and the south. The Rekambo territory in the west borders the Atlantic Ocean (for more details, see Martínez-Íñigo et al. in review).

### Data collection

Behavioral data via direct observations were collected before (2005–2016) and after the habituation of the majority of individuals (January 2017–June 2019). In addition, camera trap data from chimpanzees were collected from May 2017 to March 2019.

#### Behavioral observations

Observers followed chimpanzee parties of the Rekambo community from January 2017 to June 2019, from morning to evening (see Results for the total number of observation days and time). We observed more than one party simultaneously whenever possible. In the case of fissioning, observers stayed with the largest party if there were no observers available to follow all the parties. Whenever an intercommunity encounter occurred, observers collected information on an *ad libitum* basis (Martin and Bateson 1993) through field notes, videos (Sony Digital 4K video camera), and vocal recordings (Samsung Galaxy Xcover 3 and Cyrus CS24 smartphones). The resulting data were transcribed into detailed reports.

Based on the classification of Wilson and colleagues (2012, 2014), we categorized four different types of community encounters:

- Acoustic encounters, involving individuals of different communities that only exchanged vocalizations without visual contact (Wilson et al. 2012; Goodall 1986; Boesch and Boesch-Achermann 2000).
- Visual encounters, involving individuals of different communities seeing each other. They often exchanged noncontact aggression, such as displaying and charging. These encounters could involve the exchange of vocalizations between communities.
- Physical encounters, involving individuals of different communities using contact aggression.
- Lethal encounters, involving individuals of different communities interacting with each other and resulting in the death of at least one individual.

We classified events as acoustic intercommunity encounters if, after hearing distant chimpanzee vocalizations, individuals of the Rekambo community were vigilant, pilo-erected, ran away, showed reassuring behaviors (e.g., embracing, mounting), and charged in the direction of the vocalizations. Similar reactions were seen when distant vocalizations preceded visual intercommunity encounters but not when they preceded fusions with Rekambo community members (Wilson et al. 2012). Encounters, in which unknown chimpanzees were visible, were classified as intercommunity encounters if at least one unknown adult or adolescent male was observed in visual contact with members of the Rekambo community. By 2017, all adult and adolescent males of the Rekambo community were habituated and could reliably be identified by observers. However, many females were less habituated or not habituated, and not all observers could reliably identify them while in the forest. Consequently, the sighting of unknown males guaranteed the presence of individuals from another community, while the sighting of unknown females did not.

Observers collected GPS coordinates using handheld GPS devices (Garmin, Rhino 750) whenever they detected an intercommunity encounter.

Additionally, we report killings in which no males of other communities were observed as well as killing events witnessed before the habituation of the Rekambo community (2005–2016). However, we treat these cases separately due to the uncertainty in community identity.

#### Camera trap data

We used a camera trap network comprising 27 cameras (see Fig. S1 in ESM). Camera trap models changed over time, usually being the Bushnell Trophy Cam™ HD. We set cameras to record 1-min clips at 1-s intervals whenever movement was detected. Each camera was checked every 2–4 weeks to replace SD cards and batteries (see Arandjelovic et al. 2014 for camera trap installation and maintenance).

We compiled chimpanzee videos recorded between May 2017 and March 2019 (total monitoring time: 352,321 h). Consecutive chimpanzee videos recorded by the same camera and separated by less than 15 min were considered as one event (Head et al. 2013). Chimpanzee events were examined by 1–4 trained observers, who identified each individual whenever possible or otherwise classified them in age-sex classes following the distinction by van Lawick-Goodall (1968). Events were categorized as “foreign community” when at least one adult or adolescent male was visible that did not belong to the Rekambo community. In addition, the tag “Rekambo” was assigned whenever known members were visible in the event. No community was assigned if the event did not meet either of these two conditions. Foreign community events were further evaluated to assess whether individuals were on a territorial patrol. A territorial patrol is characterized by conspicuous behaviors such as individuals traveling silently, slowly, and close to each other, stopping to listen, and scanning the environment to gather information (Mitani and Watts 2005). We considered that a camera trap event showed a territorial patrol if it contained all these elements.

### Mapping intercommunity interactions and killings

#### Behavioral observations

**Fig. 1:**
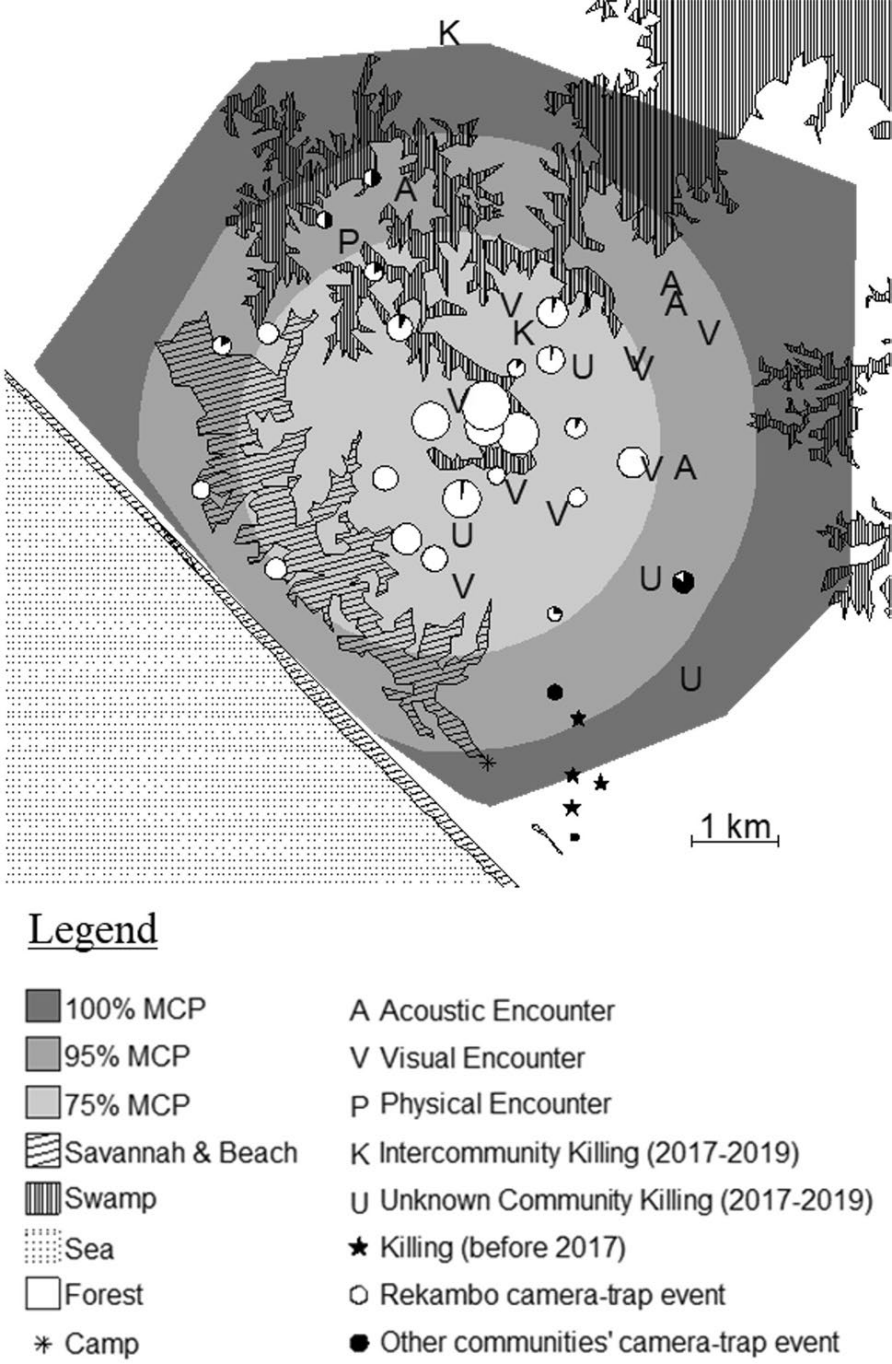
Map of the Rekambo community and overview of intercommunity encounters

We used R (v.4.0.2, R Core Team 2020) to represent the locations of the encounters on a map of the study area. We distinguished between the different intercommunity encounter types, killings before the habituation period, and killings in which the community of the victim could not be assigned (see Fig. 1). We included the home range (100% and 95% Minimum Convex Polygon, MCP) and core area (75% MCP) of the Rekambo community, as calculated in a previous study (Martinez-Íñigo et al. in review), to provide context to the data. We used MCP throughout this manuscript for comparison with other chimpanzee studies (Herbinger et al. 2001; Amsler 2009). Results based on the home range calculated with Biased Random Bridges are available in the Electronic Supplementary Material (see Results S1 and Fig. S2).

Figure 1 shows a map of the home range of the Rekambo community and locations of intercommunity encounters, killings, and camera traps. Camera traps are depicted as circles whose size is directly proportional to the number of chimpanzee events recorded by the camera, corrected for monitoring time. Colors inside the circles represent the proportion of events detected for Rekambo (in white) and other communities (in black) by each camera. Created with R (v.4.0.2, R Core Team 2020).

#### Camera trap data

The proportions of events of individuals of the Rekambo and foreign communities recorded by each camera trap were plotted as pie charts in their corresponding geographical locations (see Fig. 1). The diameter of the pie charts was proportional to the chimpanzee event capture rate (i.e., the number of chimpanzee events/camera monitoring time) of each camera. The camera monitoring time was the sum of the time elapsed between the first and last videos recorded between maintenance revisits.

### Evaluating spatial overlap between communities

To investigate whether there was temporal landscape partitioning between communities in the Rekambo core area (i.e., 75% MCP), we recalculated the MCPs for those nine mont̄hs in which sightings from other communities had been recorded within the core area from January 2017 to April 2019 (see Fig. S3 in ESM). The detailed methods can be found in Martínez̄-Íñigo and colleagues (in review).

Moreover, to evaluate the spatial overlap between the core area of the Rekambo community and sightings from other communities, we calculated a 100% MCP using 30 locations corresponding to intergroup encounters (*N* = 16) and camera traps in which sightings of other communities had been confirmed (*N* = 14). We saved the 75% MCP for the Rekambo community and the 100% MCP of other communities as shapefiles using the function *writeOGR* (package *rgdal v1.5-18*, Bivand et al. 2020). We then calculated the area exclusively used by the Rekambo community in QGIS (QGIS Development Team 2019) with these shapefiles. Finally, we repeated the process using only intercommunity encounter locations (*N* = 16) to calculate a 100% MCP for comparison with a similar study (Amsler 2009).

### Statistical analyses

We compared party sizes, the number of adult males within parties, and the chimpanzee event capture rate inside and outside the Rekambo core areas using two-tailed two-sample *t* tests or Wilcoxon rank-sum exact tests, depending on data distribution. We evaluated normal distribution using kurtosis and skewness values, accepting ± 2 as compatible with normality. Party size was defined as the total number of independent chimpanzees (i.e., adults and adolescents) from the same community observed during an intercommunity encounter (Amsler 2009). We evaluated the equality of variances using the *F* test and performed a *t* test for equal or unequal variances accordingly. *p* values lower than 0.05 were considered significant. We used R (v.4.0.2, R Core Team 2020) to perform statistical analyses.

## Results

From January 2017 to June 2019, parties of chimpanzees of the Rekambo community were followed for 831 days for a total of 8837 h. During this time, we recorded 16 intercommunity encounters on 15 different days. Four encounters were purely acoustic, nine were visual, one was physical, and two resulted in killings of two chimpanzees (see Tablē 1). On five of these encounters, the involved individuals of the Rekambo community were patrolling before the events. Fourteen camera traps detected a total of 28 events of foreign communities between May 2017 and March 2019. Thirteen events occurred within the core area of the Rekambo community (see Table S1 in ESM).

**Table 1:** Intercommunity encounters of the Rekambo community

Date	Encounter type	Location	Rekambo party (size)	Foreign party	Victim(s)
14/06/2017	Visual	Core area	4AM (4)	4AM, 2AU	–
25/07/2017	Visual	Core area	4AM, 1ADM (5)	1AM	–
30/09/2017	Visual	Core area	5AM, 1AF, 2JF (6)	9AM, 3AF	–
06/10/2017	Visual	Core area	5AM, 2AF, 1ADM, 1JF (8)	xUU	–
23/12/2017	Acoustic	Periphery	6AM, 1AF (7)	*	–
29/12/2017	Visual	Core area	7AM, 1AF, 1JF (8)	5AM, 1AF	–
11/01/2018	Visual^P^	Core area	8AM, 3AF, 4ADM, 2JF, 1JU (15)	1AM, 1AF, 1JF	–
20/01/2018	Visual	Core area	7AM, 2AF, 4ADM, 2JF (13)	5AM, 2AF, 1JU	–
20/01/2018	Acoustic	Periphery	7AM, 2AF, 4ADM, 2JF (13)	*	–
24/01/2018	Visual^P^	Periphery	8AM, 3AF, 3ADM, 2JF, 1JI (14)	2AM, 5AU	–
03/09/2018	Killing	Core area	3AM, 3AF, 1ADM, 1ADF, 1JM, 1JF (8)	1AM	AM^†^
04/10/2018	Physical	Periphery	5AM, 3AF, 2ADM, 1ADF, 1JF, 1JM, 1JU (11)	1AM, 1AF, 1ADM, 1ADF, 1JF, 1UU	ADM, ADF
23/11/2018	Acoustic^P^	Periphery	5AM, 3AF, 3ADM, 2ADF, 1JM, 1IF (13)	*	–
06/01/2019	Visual	Core area	5AM, 4AF, 3ADM, 1ADF, 2JM, 1IF (13)	5AM	–
17/05/2019	Acoustic^P^	Periphery	4AM, 1AF, 1ADF (6)	*	–
17/06/2019	Killing^P^	Out of range	6AM, 3AF, 3ADM, 2ADF, 1JM, 1IF (14)	1AM, 1AF, 1IF, 1UU	AF, IF^†^

Table 1 shows an overview of intercommunity encounters between the Rekambo community and neighboring communities from January 2017 to June 2019 as a function of date, encounter type, location, Rekambo party and size, foreign party and victim(s). The “date” column contains the specific day of each encounter depicted as “day/month/year”. The “encounter type” column classifies the encounter in one of the four considered categories: (1) Acoustic encounter, (2) visual encounter, (3) physical encounter, or (4) lethal encounter. The superscript P indicates that members of the Rekambo community patrolled before the encounter. The “location” column refers to where within the home range of the Rekambo community (January 2017–April 2019) the encounter took place. The location categories are as follows: Core area: within 75% MCP; periphery: 75–95% MCP; out of range: outside 95% MCP. The “Rekambo party” column refers to the number and age-sex classes of the community members seen during the encounter, with the size of the party given in brackets. The “foreign party” column refers to the number and age-sex classes of the individuals of other communities observed during the respective encounter. The “victim(s)” column refers to the age-sex class of the individuals of other communities that were physically aggressed (e.g., bitten, hit, grabbed) during the respective encounter. If the attack was lethal, it is depicted via the superscript^†^. The age-sex classes of individuals in the “Rekambo party”, “foreign party”, and “victim(s)” columns were labeled as follows: number of individuals + age category + sex. *A* adult, *AD* adolescent, *J* juvenile, *I* infant, *F* female, *M* male, *U* unknown. *x* unknown number

*Not observed

^–^Not applicable

Additionally, we recorded the killing of four infants and a juvenile male whose communities could not be determined. One such killing was preceded by a territorial patrol (see Table 2). Finally, between 2005 and 2016, five possible cases of intercommunity killings were recorded in the area of study (see Table 3).

**Table 2:** Lethal interactions with individuals of unknown community membership

Date	Location	Rekambo party	Encountered party	Victim(s)	Cannibalism
24/12/2017	Core area	6AM, 1AF, 1ADM, 2JF	Not observed	IU^I†^	Yes
17/02/2018	Periphery	7AM, 2ADM, 1JF	2AF, 1JM, 1 IU	2AF, JM^O†^, IU^I†^	Yes, Yes
07/05/2019	Core area	2AM, 2AF, 1ADM, 1 IU	1AF, 1JM, 1 IU	AF, IU^O†^	Yes
23/06/2019	Out of range^P^	7AM, 3AF, 3ADM, 2ADF, 2JM, 1IF	1AF, 1IF	AF, IF^O†^	No

Table 2 depicts lethal interactions with individuals of unknown community membership led by the Rekambo community from January 2017 to June 2019 as a function of date, location, Rekambo party, encountered party, victim(s) and cannibalism. The “date” column contains the specific day of each encounter depicted as “day/month/year”. The “location” column refers to where within the home range of the Rekambo community (January 2017–April 2019) the encounter took place. The location categories are as follows: Core area: within 75% MCP; periphery: 75–95% MCP; out of range: outside 95% MCP. The superscript P indicates that individuals of the Rekambo community patrolled before the encounter. The “Rekambo party” column refers to the number and age-sex classes of the community members seen during the whole encounter. The “encountered party” column refers to the number and age-sex classes of the individuals of unknown communities observed during the encounter. The “victim(s)” column refers to the age-sex class of the individuals of unknown communities that were physically aggressed (e.g., bitten, hit, grabbed) during the encounter. If the attack was lethal, the individual who was killed has the superscript †. The superscripts I and O indicate the kind of evidence available for the event: O = observed event: Observers witnessed how the victim was killed; I = inferred: The individual killed was found dead during the encounter with visual signs (e.g., chimpanzee teeth wounds) of having been killed by chimpanzees. Age-sex classes of individuals in the “Rekambo party”, “encountered party”, and “victim(s)” columns were labeled as follows: number of individuals + age category + sex. *A* adult, *AD* adolescent, *J* juvenile, *I* infant, *F* female, *M* male, *U* unknown. The “cannibalism” column refers to whether individuals of the Rekambo community fed on the remains of the killed victims

**Table 3:** Chimpanzee intraspecific killings before 2017

Date	Observation type	Location	Chimpanzees observed	Victim	Cannibalism
08/06/2006	Inferred	Out of range	4AM, 1AF, 3AU, 1JU	JU	*
10/10/2006	Inferred	Out of range	1AF, 2AU	AM	*
23/06/2007	Inferred	Out of range	1AM, 1AF, 5AU, 1IU	IU	Yes
16/12/2008	Suspected	Periphery	3AM, 1AF, 3AU	AM	*

Table 3 depicts chimpanzees’ intraspecific killings before the habituation of the majority of individuals of the Rekambo community as a function of date, observation type. location, chimpanzees observed, victim, and cannibalism. Date is depicted as “day/month/year”. “Observation type” is categorized following the distinctions by Wilson and colleagues (2014). Inferred: Aggression witnessed, corpse or parts found after the aggression, and traces indicating intercommunity attack found. Suspected: Body found showing injuries compatible with intraspecific coalitional aggression. “Location” refers to where within the home range of the Rekambo community (January 2017–April 2019) the encounter took place. Periphery: 75–95% MCP; out of range: outside 95% MCP. “Chimpanzees observed” refers to the number and age-sex class of the attackers (unknown community membership). Individuals were labeled as follows: Number of individuals + age category + sex. *A* adult, *AD* adolescent, *J* juvenile, *I* infant, *F* female, *M* male, and *U* unknown. The asterisk (*) refers to “not observed”

### Presence of other communities in the Rekambo home range detected by camera traps

Between January 2017 and March 2019, the camera traps recorded a total of 682 chimpanzee events: 492 events (72.14%), 28 foreign community events (4.10%) (see Table S1 in ESM), and 162 events (23.75%) for which we could not assign a community. The chimpanzee event capture rate was higher inside the Rekambo core area than outside (Wilcoxon rank-sum test, W = 128, *p* value < 0.01). The capture rate of foreign community events in cameras outside the Rekambo core area was higher than in cameras placed inside the Rekambo core area (Wilcoxon rank-sum test, W = 31; *p* value < 0.05).

Foreign community events recorded within the core area of the Rekambo community and outside showed a similar number of independent individuals (Wilcoxon rank-sum test, W = 127.5, *p* value = 0.16) and adult males (Wilcoxon rank-sum test, W = 134, *p *value = 0.08). Adult females were involved in five of 13 foreign community events recorded inside the Rekambo core area and nine of 15 events recorded outside the core area. Juveniles and infants were detected in a similar proportion of events in both areas (core area: Six of 13 events; outside core area: Seven of 15 events). Five events showed behaviors characteristic of territorial patrols (see Table S1 in ESM). Two of those events contained adult females with dependent offspring. Adolescent females participated in four of the five recorded patrols.

Concerning the 13 foreign community events detected inside the core area (see Table S1 in ESM), six events occured within the monthly core area (see Fig. S3 in ESM).

### The overlap between the Rekambo core area and sightings of other communities

The exclusive core areas were 2.69 km^2^ and 9.52 km^2^ when subtracting the 100% MCP generated with locations of all sightings of foreign communities (i.e., camera traps plus encounters) and 100% MCP of the encounters, respectively (see Fig. S4 in ESM). These were 14.31% and 50.64% of the core area (18.80 km^2^) and 4.56% and 16.13% of the Rekambo 100% MCP (59.03 km^2^).

### Intercommunity encounters

Intercommunity encounters occurred 0.53 times per month, or on 1.92% of the days when observers followed parties of the Rekambo community. The party size (see Table 1) was similar in encounters inside and outside their core area (Wilcoxon rank-sum test, W = 21.5, *p* value = 0.31] as was the number of adult males [*t* (11.73) = 0.05, p = 0.96]. Adult males were present in all 16 encounters, and adult females were present in twelve encounters. Territorial patrolling was observed prior to five of the 16 recorded encounters. All patrols included adult females, and four included juveniles and/or infants (see Table 1).

Purely acoustic encounters (four of 16 encounters) happened beyond the core area of the Rekambo home range. In all of them, the first reaction of the Rekambo chimpanzees was to move towards the vocalizations and sounds of individuals of other communities. However, in one case, individuals of the other community appeared to retreat. “Appearing to retreat” refers to observers having the impression that the vocalizations and drums of chimpanzees from other communities came progressively from longer distances. In contrast, the Rekambo chimpanzees remained still or moved towards the vocalizations and drums. In two other acoustic encounters, the Rekambo members withdrew, moving away from the direction from where the vocalizations of individuals of other communities were heard. In the remaining case, it was uncertain which individuals retreated.

Visual encounters accounted for nine of the 16 encounters observed and always involved noncontact aggression (e.g., charges, chases, and displays). Most of these events took place inside the Rekambo core area (eight of the nine visual encounters; see Table 1). In the periphery, the other community initiated the only visual contact by approaching members of the Rekambo community. In the core area, individuals of the Rekambo community approached the intruders in three cases, while individuals of the neighboring community approached Rekambo in four cases. In the remaining case, it was unclear which individuals initiated the event. In all visual encounters, individuals of the other community retreated, presumably after seeing human observers. Adult females of the Rekambo community were present in seven visual encounters and participated in noncontact aggression in three of them. One suspected intercommunity copulation occurred in one of the visual encounters.

We confirmed only one physical encounter, which occurred in the periphery of the Rekambo territory. The aggression consisted of coalitional aggression between seven individuals of the Rekambo community (four adult males and three adult females) against an adolescent male after most of his accompanying community members had escaped. In addition, at least one adult female of the Rekambo community attacked an adolescent female from the other community during the same event. The observers suggested that this attack started when the adolescent female had tried to support the adolescent male that had been attacked by the Rekambo party. Both victims escaped.

There were two lethal encounters with two victims: an adult male, and an infant female (see Table 1). Thus, the annual intercommunity killing rate between January 2017 and June 2019 was 0.8 individuals/year. Nine members of the Rekambo community, including three adult females, an adolescent female, and a juvenile male, participated actively in killing the adult male, bitting, kicking, and hitting him, dragging him around, and jumping on him. The attack lasted for over 20 min, although the observers of this event thought that the attacked male was already dead after 15 min of the aggressions. In addition, at least two adult males, one adult female, and one adolescent female bit off pieces of the victim's body. A different adolescent female licked the blood of the victim of her fingers. Observers examined the body the day after the killing. The most severe injury was a deep cut on the abdomen from which the intestines protruded. Further injuries were located on the left armpit, the throat, the chest, and all limbs.

The killing of the infant female was preceded by a territorial patrol formed by six adult males, three adult females, three adolescent males, two adolescent females, a juvenile male, and an infant of the Rekambo community. After 1 h 43 min, the party found an adult male, an adult female carrying an infant female, and at least one more unknown individual. The chimpanzees from the other community tried to flee but the Rekambo chimpanzees caught the adult female with her infant. They attacked her and her infant until they could separate them. Then the Rekambo chimpanzees concentrated the attacks on the infant. First, the foreign adult female left, and then two of the Rekambo adult males grabbed the infant and pounded her against the ground and against trees. The entire encounter lasted around seven min. The victim was not cannibalized.

### Lethal interactions with individuals of unknown community membership

Between January 2017 and June 2019, chimpanzees of the Rekambo community killed four infants and a juvenile whose community membership could not be confirmed due to the absence of adult or adolescent males accompanying the victims (see Table 2).

In the first case (24/12/2017, see Table 2), observers did not witness the aggression. They found an adult male from the Rekambo community with a the body of dead infant in his hands. Before this, the Rekambo community party had run towards that location while vocalizing. After this, an adult male, different from the one holding the dead infant, bit into its right leg, tore a piece of flesh, and chewed on it. He then bit into the dead infant's right arm without tearing the flesh.

In the second case (17/02/2018), a party of Rekambo chimpanzees (see Table 2) heard pant-hoots and ran towards their origin. They found two unidentified adult females with an infant and a juvenile male (02:48 pm). The unidentified individuals unsuccessfully attempted to escape. The Rekambo adult males blocked them and attacked the juvenile while the unidentified females attempted to protect him. Amid the attacks, observers discovered a dead infant on the ground. One of the Rekambo adult males took the infant's corpse. Subsequently, another male took it from him. One of the unidentified females charged the male holding the infant, took the corpse, and ran back to the other female to guard the juvenile. Both females defended their supposed offspring aggressively by charging and even biting the Rekambo males. The Rekambo males were attacking the juvenile male and attempted to retrieve the infant's corpse. The female, carrying the infant's corpse, sustained large lacerations to her right shoulder and on the right side of her lower back. At 02:56 pm, an adult male took the infant's corpse after it had been passed around between three more adult males and a juvenile female from the Rekambo community. They often carried the infant's corpse in their mouth while moving around and attacking the females and the juvenile. At least two of the males and the juvenile female fed on the infant. The supposed mother of the infant presented her vulva to two of the adult males but no copulations were observed. When the Rekambo chimpanzees had the infant's corpse in permanent posession, they started traveling while forcing the unidentified juvenile and his supposed mother to accompany them. The other unidentified female followed them for some minutes but then left without the Rekambo chimpanzees showing any resistance (03:17 pm). The attacks on the juvenile male became now more frequent and violent.

The remaining unidentified female attempted to protect her supposed juvenile and was charged several times until she went out of the observers' view at 03:30 pm. The males continued attacking the juvenile. One of them hit him with a branch, tore a piece of flesh out of the juvenile's leg, and ate it. The juvenile female of the Rekambo community used her fingers and leaves to collect blood from the unidentified juvenile and lick it. The Rekambo party ceased the attack on the juvenile chimpanzee at 05:15 pm. The juvenile was, at this point, still alive but seemed lethargic, unable to move, and severely injured. Observers found his corpse the following day at the same location where the Rekambo party had left him.

In the third case (07/05/2019), a party of Rekambo chimpanzees (see Table 2) encountered three individuals passing by, one unidentified adult female with an infant and one juvenile chimpanzee. Two of the Rekambo males responded to the party by chasing the unidentified female for ~30 min. While one of the males attacked the female, the other grabbed her infant and ripped open its abdomen with a single bite. The female attacked the male, who had aggressed her. He retaliated by injuring her before allowing the female to leave with the juvenile without any more interactions. The two males fed on the infant's corpse until only the thorax and one hand remained.

The fourth attack (23/06/2019) was observed beyond the usual home range of the Rekambo community (95% MCP, see Fig. 1) after one hour of patrolling. The party of Rekambo chimpanzees (see Table 2) ran and encircled an unidentified adult female with an infant. The observers could not observe the aggressions themselves but reported an adult male leaving the circle while holding an infant female. He subsequently started to pound her against nearby trees. The unidentified female reclaimed her infant but was again attacked until the same male grabbed the female infant from her and again pounded the infant against surrounding trees. Once the infant was dead, some of the Rekambo chimpanzees inspected it before continuing with other activities without interacting with the corpse or her supposed mother any further.

### Killings before the habituation of the Rekambo community

Before the habituation of the Rekambo community (2005–2016), the contact time with chimpanzee parties was 1060 h in total. During this period, observers recorded five inferred or suspected killings (*sensu* Wilson et al. 2014, see Table 3). The first one, recorded in August 2005, has already been described by Boesch and colleagues (2007) and will not be further discussed here. However, we report four additional events for the first time: Three inferred killings and one suspected one (see Table 3). The observers inferred three killings through direct observations or signs of aggressions between chimpanzees, followed by detecting a corpse or smaller remains (e.g., a foot). In these three cases, there was indirect evidence of intercommunity aggression, such as traces of chimpanzees leaving the place of the attack in different directions. Cannibalism was only observed in the case of an infanticide (see Table 3). The fourth case, classified as suspected, involved the discovery of the corpse of an unidentified adult male. The body showed injuries compatible with an intraspecific coalitional attack. However, it is uncertain whether it was caused by an intra- or intercommunity aggression. In contrast to the other three cases, there was no indirect evidence indicating an intercommunity attack.

## Discussion

Here, we provide the first insights into intercommunity interactions of a community of central chimpanzees living in the Loango National Park, Gabon, after the habituation of the majority of its members. We used a combination of direct observations and camera trap data collected over a period of two years and six months. We detected neighboring communities in most of the Rekambo core area with little evidence of temporal landscape partitioning between communities. Encounters occurred less than once per month. The most frequent encounter type was visual, followed by acoustic, lethal, and physical encounters (Wilson et al. 2012, 2014). In addition, individuals of the Rekambo community killed four infants and one juvenile chimpanzee of unknown community membership during the same period. Adult males were the main participants in nonphysical and physical aggression, but adult females were also involved. Adult females of both the Rekambo community and other communities participated in territorial patrols, at times accompanied by dependent offspring. We also documented four new cases of intraspecific killing before the habituation of the community (2006–2016).

### Presence of other communities inside the Rekambo home range

The rates of detecting foreign communities by camera traps and intercommunity encounters were higher in the periphery of the Rekambo home range than inside the Rekambo core area. These findings are in line with observations of intercommunity encounters at other chimpanzee study sites (Wilson et al. 2004, 2007, 2012; Boesch et al. 2008).

The proportion of territory exclusively used by individuals of the Rekambo community (4.56% estimated using camera trap data plus encounters; 16.13% using encounters only) was similar to results reported for the Taï communities (4–14%, Herbinger et al. 2001) but lower than those documented at Ngogo, Kibale National Park, Uganda (23%, Amsler 2009). Intercommunity encounters in chimpanzees cause severe injuries and death more frequently than in other nonhuman primate species (e.g., Crofoot and Wrangham 2010). Therefore, chimpanzees are expected to have negligible home range overlap (Wrangham et al. 2007). Spatial overlap may occur without temporal overlap reducing encounter chances. For example, the communities M and K at Mahale (Tanzania) have been reported to monopolize the overlapping area of their ranges in different seasons (Nishida 1979). However, our camera trap data revealed that other communities visited areas that individuals of the Rekambo community also frequently used at the same time (see Fig. S3 in the ESM). Hence, it seems that temporal landscape partitioning between communities does not occur at our site.

Individuals of other communities entered the core area of the Rekambo community during deep incursion patrols. Incursions into the core area of other communities have been suggested to precede territory expansions and group extinctions at other long-term study sites (e.g., Nishida et al. 1985; Goodall 1986; Mitani et al. 2010). Earlier camera trap studies at our site detected a relatively low degree of ranging overlap between neighboring communities (Head et al. 2013). Furthermore, they revealed the existence of up to nine adult males belonging to the Rekambo community that were no longer present in our study period (Estienne et al. 2017). Consequently, the present study may portrait a period of unusually intense territorial behavior.

However, the low intercommunity encounter rate may argue against such an interpretation. Nevertheless, several Rekambo infants and juveniles disappeared during the study period considered here. Moreover, in August 2018, we found one of the Rekambo adult males severely injured with wounds typically caused during intraspecific coalitional attacks (e.g., removal of one testis). Thus, individuals of the Rekambo community may experience intercommunity encounters when not being followed by human̄ observers. This explanation might also explain the discrepancy between the low intercommunity encounter rate and the increased level of intercommunity intrusions as seen with camera traps compared to the study by Head and colleagues (2013).

### Intercommunity encounters

The Rekambo community experienced intercommunity encounters less often than most other chimpanzee communities with unhabituated neighbors from other long-term research sites (0.53 times per month, or in 1.92% of the observation days). For example, researchers documented 1.48–3.75 encounters per month at Taï, Mahale, and Ngogo (Boesch et al. 2008), and in 5.5% of the follows at Gombe, Tanzania (Goodall 1986). Similarly to Rekambo, the Kanyawara community (Kibale National Park, Uganda), which has a lower rate of encounters than Rekambo (1.9% of the follows, Wilson et al. 2012), also lacks neighbors at one side of their territory. Individuals of the Rekambo community spent considerable amounts of time on the side of their territory with no neighbors (see the coastal core areas in Fig. S2 in ESM). Thus, the lack of neighbors in the area plus the proportion of time spent there may contribute to the relatively low intercommunity encounter rate.

Studies on other chimpanzee communities consistently found that acoustic encounters were the most common encounter type, followed by visual, physical, and in some cases, lethal events (Watts and Mitani 2001; Boesch et al. 2008; Wilson et al. 2012). In contrast, visual encounters were the most frequent in our community, followed by acoustic, lethal, and physical encounters. A potential explanation for the low rate of acoustic encounters may be that observers were not yet able to reliably identify and report acoustic encounters. Alternatively, the excellent visibility in Loango in contrast to other sites (e.g., Taï National Park; Boesch et al. 2008) may facilitate visual contact. Moreover, and in contrast to findings from other sites (Goodall 1986; Boesch and Boesch-Achermann 2000; Watts and Mitani 2001), individuals of the Rekambo community and neighboring communities seemed willing to establish visual contact. Studies in other communities found that larger parties with more males are more prone to approach and attack foreign parties (Watts and Mitani 2001; Wilson et al. 2012). Since observers tended to stay with the largest party, consisting primarily of males due to their higher levels of habituation, their presence may have influenced the rates of agonistic behaviours observed.

Furthermore, our results showed that the party size of the Rekambo community and the number of males within the party were similar in encounters inside and outside the core area. However, parties tended to be larger and included higher numbers of males in the periphery than reported at other study sites (Mitani and Watts 2005; Wilson et al. 2007, 2012). Again, this result could be due to the observers' tendency to follow the largest party present. Nevertheless, given the low proportion of exclusive home range of the Rekambo community (see Fig. S4 in ESM), members might regularly travel in larger parties for protection.

### Participation of females with dependent offspring in patrols

Adult females from the Rekambo community and other communities participated in territorial patrols and were often accompanied by their dependent offspring. These results are in line with observations at Taï, where females were involved in 57% of all observed patrols (Boesch and Boesch-Achermann 2000), and were also frequently accompanied by their dependent offspring (see Movie S1 in Samuni et al. 2017). However, at most other long-term sites, territorial patrols are a predominantly male activity (Reynolds 2005; Mitani and Watts 2005; Gilby et al. 2013). The presence of dependent offspring in patrols is surprising, given the high infanticide risk during encounters (Newton-Fisher 1999; Watts et al. 2002; Wilson et al. 2004, present study). Since individuals and parties of other communities enter the core area of the Rekambo community, females with dependent offspring might be safer joining patrols with many males than staying in the core area in smaller parties without males.

The tendency of females of our community to join territorial patrols may suggest that central chimpanzees are similar to western chimpanzees at Taï in their propensity to form bisexually bonded communities (Lehmann and Boesch 2005).

### Females as attackers and victims during intercommunity encounters

The Rekambo community females participated in noncontact and contact aggression during intercommunity encounters, including killing an adult male. Female involvement in chimpanzee intercommunity aggression is rare at most sites but seems to be a substantial aspect at Taï (see Furuichi 2020 for a review).

In the only confirmed intercommunity infanticide, individuals of the Rekambo community attacked the mother until they were able to obtain her infant. Subsequently, the mother was ignored. Similar attacks on females with dependent offspring were reported at the Sonso community from the Budongo Forest, Uganda (Newton-Fisher 1999), and the M-group at Mahale (Kutsukake and Matsusaka 2002). However, at Ngogo and Gombe, attacks on females, sometimes lethal, continued even after their infants had been obtained (Watts and Mitani 2000; Wilson et al. 2004, 2014). In contrast, at Taï, infanticides, and attacks on extra-community females were very rare (Boesch et al. 2008; but see Wilson et al. 2014).

### Lethal intercommunity aggressions

The rate of intercommunity killings by individuals of the Rekambo community (0.8 killings/year) was higher than that documented for all other long-term chimpanzee communities studied except for Ngogo (1.38 killings/year, see Wilson et al. 2014 for comparative data on intercommunity killings across *Pan* communities and the raw data from which the killing rate for Ngogo was calculated). Moreover, if all the killings, for which we could not infer community membership of the victims, were not caused by individuals of the Rekambo community, the intercommunity killing rate would increase to 2.8 individuals/year thereby largely surpassing the rate reported for the Ngogo community (see discussion below).

In a cross-site comparison, Wilson and colleagues (2014) found that killing rates were positively correlated with the number of community males and population density. During the study period considered here, the Rekambo community had seven to eight adult males and showed a density of 0.77–1.11 chimpanzees/km^2^ (Martinez-Íñigo et al. in review). According to the study of Wilson and colleagues (2014), the following four communities had the closest number of males and population density values to the Rekambo community: Taï East (4.90 males, 1.4 chimpanzees/km^2^), Taï South (5.30 males, 1.6 chimpanzees/km^2^), Fongoli (Fongoli, Senegal, 11.80 males, 0.37 chimpanzees/km^2^), and Moto (Goualougo Triangle, Republic of Congo, 11.00 males, 1.7 chimpanzees/km^2^). Of these communities, intercommunity killings were only reported for the Taï South community, with a killing rate of 0.04 killings/year (Wilson et al. 2014). Thus, we documented an intercommunity killing rate much higher than expected based on the number of males living in the Rekambo community and population density.

It may be possible that the Rekambo community was experiencing a temporarily higher rate of killings. For example, at Gombe, researchers reported comparable killing rates after a community fission (Goodall 1986). However, our long-term data do not suggest that a community fission occurred during our study period. Adult males and females which disappeared between 2009–2014 (Head et al. 2013; Estienne et al. 2017) did so progressively over the years instead of all individuals disappearing at once, as expected in community fission. Moreover, none of them has been detected on camera trap footage in the periphery of the Rekambo territory, which would have been probable in the case of community fission.

We cannot rule out that the killing rate of reported here for the Rekambo community was temporarily high (as reported at Gombe) for any other reason. Nonetheless, between July 2019–September 2020, individuals of the Rekambo community killed three additional individuals of other communities (Loango Chimpanzee Project, unpublished data). Consequently, a high lethality could be a permanent feature of the intercommunity relationships at Loango rather than representing a temporary pattern. If this explanation is true, intense intercommunity competition might be driven by consistent ecological factors. For example, chimpanzees at Loango might experience intense interspecies competition with western lowland gorillas (*Gorilla gorilla gorilla*), as well as forest elephants (*Loxodonta cyclotis*). The evidence thus far shows dietary overlap among the three species as well as signs of interspecies competition (Martinez-Íñigo et al. in review; Southern et al. in review; Head et al. 2012). However, we currently lack the systematic data to evaluate the extent and impact of interspecific competition occuring at Loango.

### Lethal interactions with individuals of unknown community membership

Between January 2017–June 2019, chimpanzees from the Rekambo community were observed and inferred to kill four infants and a juvenile whose community could not be assigned (see Table 2). Intracommunity killings of unweaned individuals have been reported in as many communities as intercommunity killings (Wilson et al. 2014). The behavior of killers and victims was not remarkably different in intra- and intercommunity infanticides. In both, cannibalism is frequent but not universal. For instance, mothers might continue to be aggressed after they lose hold of their offspring, but not mandatorily. Intra- and intercommunity infanticides can be conducted by coalitions of females, males, or mixed parties. Both types of attacks are frequent in eastern chimpanzees but seem to be absent in western chimpanzees (Arcadi and Wrangham 1999; Lowe et al. 2020). The most consistent difference between intra- and intercommunity infanticides is the average age of victims (Wilson et al. 2014; Lowe et al. 2020). Intracommunity infanticides tend to target infants younger than one year of age. They are often the offspring of low-ranking mothers whose mating efforts have not yet concentrated towards the highest-ranking males of the community (Nishida and Kawanaka 1985; Hamai et al. 1992).

The age of three of the victims killed by individuals of the Rekambo community could not be evaluated accurately. The ages of the other two were estimated as two and four years of age, respectively. They were above the average age range of intracommunity killings but within the observed age range reported in the study by Wilson and colleagues (2014). The location of the attacks does not reliably reflect whether the attacks occurred within or between communities. Three victims were killed within the core area of the Rekambo community and two victims were killed in the periphery. However, since most confirmed intercommunity encounters occurred within the core area, and the Rekambo females are known to patrol with their offspring in the periphery of the territory, intra- and intercommunity infanticides could happen at any location within the home range.

It is possible that victims, whose community could not be assigned, were indeed from the Rekambo community. They could be the offspring of not yet habituated females. Such females probably only spent a minimal amount of time with our high-ranking and fully habituated males since researchers often accompanied these males and scare the females. Therefore, these unhabituated females would have a decreased chance of bonding and mating with high-ranking community males, resulting in a higher chance of intracommunity infanticide (Nishida and Kawanaka 1985; Hamai et al. 1992). Indeed, in all of these infanticides at least one of the male perpetrators was among the highest-ranking males in our community. Intracommunity infanticides in these contexts are hypothesized to coerce females to concentrate future matings into high-ranking males (Nishida and Kawanaka 1985).

If all five victims were indeed members of the Rekambo community, the intracommunity infanticide rate would be two individuals/year. This rate is much higher than that reported for the Sonso community, which showed the highest rate so far reported across all long-term study sites compared (0.35 individuals/year; Wilson et al. 2014). If, on the contrary, all infanticides were intercommunity killings, the intercommunity infanticide rate for Rekambo would be 2.4 individuals/year, and the total intercommunity killing rate would be 2.8 individuals/years (previous maximums as for data in Wilson et al. 2014: intercommunity infanticide: 0.89 individuals/year, Ngogo community. Intercommunity killing rate: 1.39 individuals/year, Ngogo community). Any value in between, with some of the killings being intracommunity and the others intercommunity, would still rank Rekambo as one of the most lethal chimpanzee communities studied so far.

### Concluding remarks and future research

Our study showed that the intercommunity encounters of individuals and parties of the Rekambo community revealed some differences to those reported from other chimpanzee communities. Visual encounters were more frequent than acous̄tic encounters and there were more killings than physical encounters. Females participated in aggression towards other communities, including a killing. Moreover, adult females joined territorial patrols while being accompanied by their dependent offspring. In addition, between January 2017–June 2019, the Rekambo community showed the second highest annual rate of confirmed intercommunity killings so far reported despite having a low population density and an average number of adult males in the community. This rate might be even higher if any of the victims of unknown community membership could be assigned to the neighboring communities.

Overall, our data suggest that between-community competition was exceptionally high at our site, at least during the study period considered here. Systematic monitoring of camera trap data will provide information on the size, composition, and ranging patterns of neighboring communities, which will aid in understanding these patterns in more detail. Furthermore, the improved habituation of the Rekambo community is enabling the collection of information on a more representative set of party sizes and compositions, which will aid in confirming or disproving the tendencies observed. Finally, future systematic research addressing the competitive interactions between chimpanzees, gorillas, and elephants may shed light on the high intercommunity competition in chimpanzees at Loango.

## Supplementary Information

Below is the link to the electronic supplementary material.

Supplementary file1 (DOCX 2240 KB)

## Data Availability

Data and material will become available at the 1000PAN database (https://www.comparative-biocognition.de/1000pan/about-1000pan). Meanwhile, they can be requested at the same source.

## Code availability

Custom mapping R code available in the Electronic Supplementary Material.
