# Supplementary material for: Intercommunity interactions and killings in central chimpanzees (Pan troglodytes troglodytes) from Loango National Park, Gabon
Source: Primates. 2021 Jun 17;62(5):709–22. doi: 10.1007/s10329-021-00921-x (PMC8410688; doi:10.1007/s10329-021-00921-x)
Supplement: Supplementary file 1 — Supplementary file1 (DOCX 2240 KB) [file 10329_2021_921_MOESM1_ESM.docx]

**Supplementary information**

**Intercommunity interactions and killings in central chimpanzees (*Pan troglodytes troglodytes*) from Loango National Park, Gabon**

**Journal:** Primates

Laura Martínez-Íñigo, Pauline Baas, Harmonie Klein, Simone Pika, Tobias Deschner

***Corresponding author:***

Laura Martínez-Íñigo

Email: laura_m_innigo@live.com

**SUPPLEMENTS**


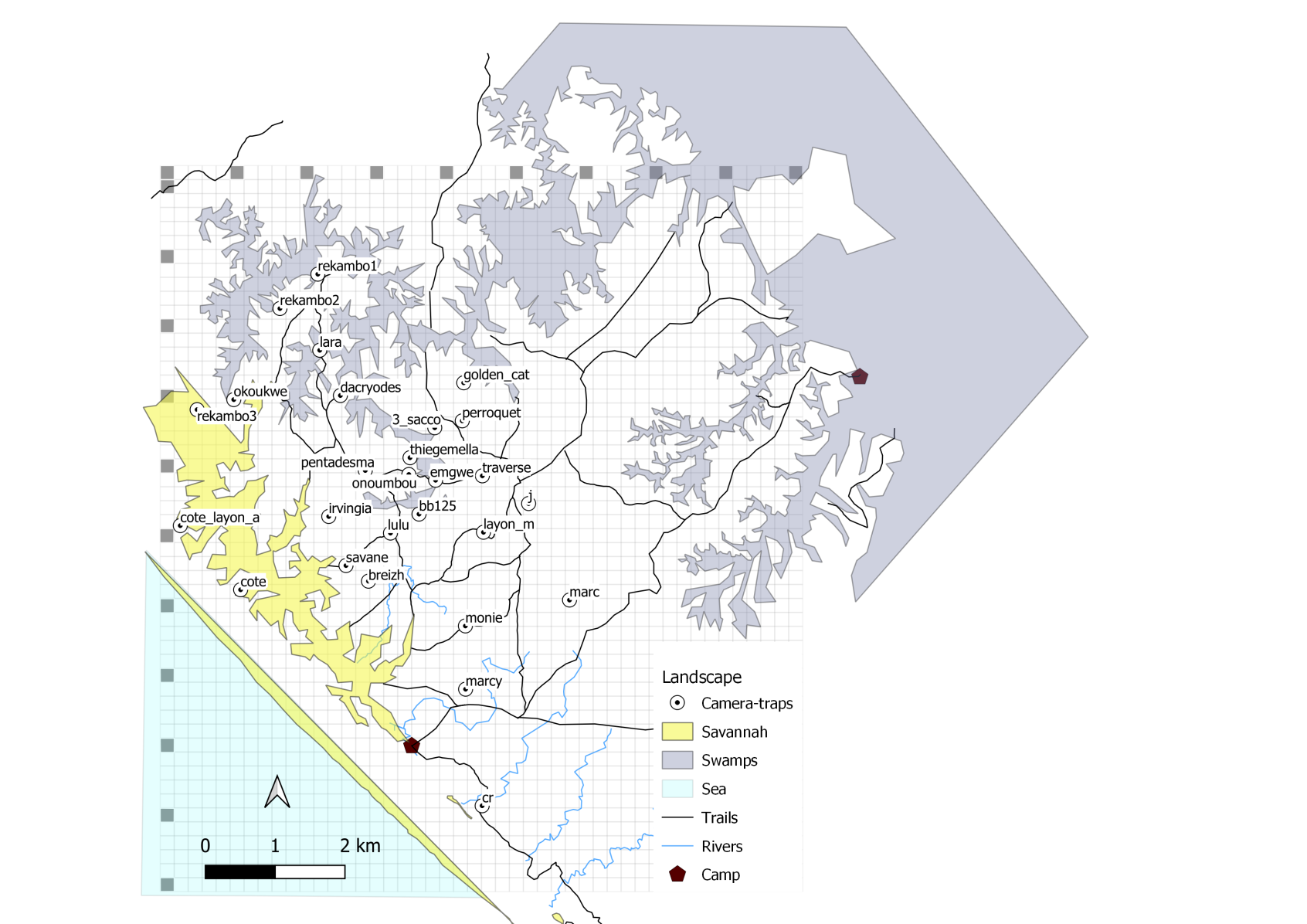


**Fig. S1** **Study site map showing the location of the camera-traps used during the study and the location of the Ozouga Research Camp.** The location of the camera-traps are depicted with a white circle with a black dot in the middle. Each camera is labeled with its name. The savannah and beach are depicted in yellow. Swamps are painted in grey. The sea is colored in blue. Trails are indicated with black lines and rivers with blue lines. The location of the Ozouga Research Camp is signaled with a brown pentagon. Created with QGIS 3.10.5-A Coruña (QGIS Development Team 2019)

**Results S1 Camera-trap and intergroup encounter results based on the Biased Random Bridges (BRB) home range**

Details on how we calculated BRB can be found in Martinez-Inigo et al. (in review).

***Presence of other communities in the Rekambo home range detected by camera-traps***

The chimpanzee event capture rate was higher inside the Rekambo core area than outside (Wilcoxon rank-sum, W = 134, p-value= 0.01289). On the other hand, the event capture rate of individuals of foreign communities was higher on cameras outside than inside the Rekambo core area (Wilcoxon rank-sum; W = 29; p-value = 0.00312).

Foreign community events recorded within and outside the core area of the Rekambo community tended to show a similar number of individuals (Wilcoxon rank-sum; W = 93, p-value = 0.7265) and adult males (Wilcoxon rank-sum; W = 102, p-value = 0.4044). Adult females were seen in 22.22% of foreign community events inside the Rekambo MCP core area and 63.15% of the events outside. Juveniles and infants were seen in a similar proportion of events in both areas (Core area: 44.44%; outside core area: 47.37%).

***Overlap between Rekambo core area and sightings of other communities***

The exclusive core area for BRB was 0.70 km^2^ and 5.34 km^2^, subtracting the 100% MCP generated with all locations where other communities had been detected and the 100% MCP of the encounters, respectively. These were 5.23% and 39.97% of the 75% BRB (13.36 km^2^), and 2.47% and 18.88% of the 95% BRB (28.28 km^2^).

***Intercommunity encounters***

Controlling for the time spent inside and outside of the 75% BRB area, the Rekambo community encountered other communities 0.0012 times/hour and 0.004 times/hour, respectively in each zone.

Rekambo party size (see Table 2) was similar during encounters inside and outside their core area (Wilcoxon rank-sum test, W = 23.5; p-value = 0.39), as was the number of adult males (t (13.98) = 0.14, p=0.89).

**References**

Martinez-Iñigo L, Baas P, Klein H, et al (in review) Home range size in central chimpanzees (*Pan troglodytes troglodytes*) from Loango National Park, Gabon


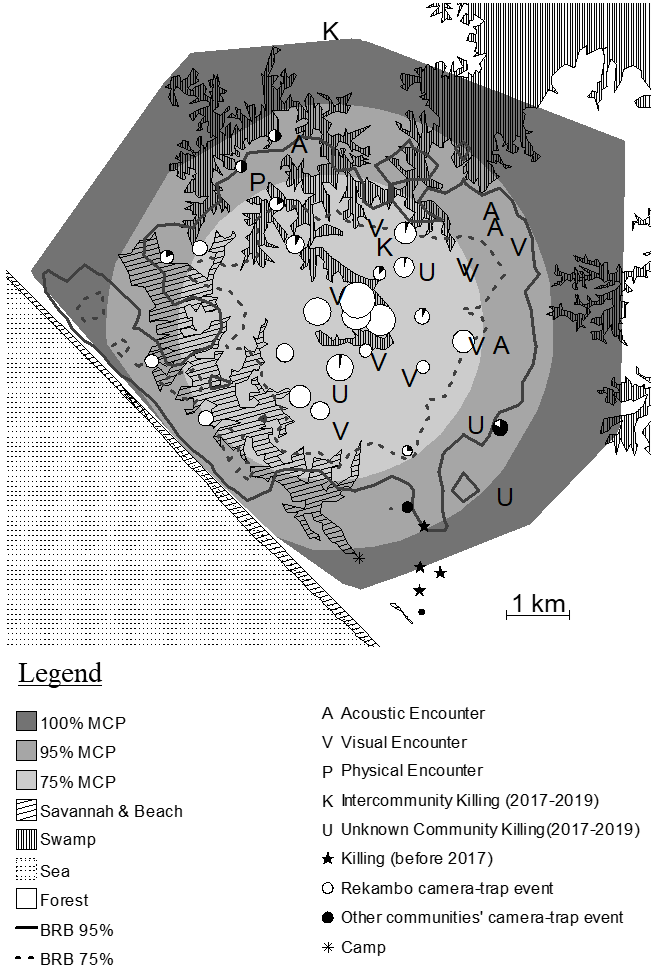


**Fig. S2 Map of the Rekambo home range (Minimum Convex Polygon, MCP, and Biased Random Bridges, BRB), location of intercommunity encounters, killings and camera-traps** Camera-traps are depicted as circles whose size is directly proportional to the number of chimpanzee events recorded by the camera, corrected for its monitoring time. Colors inside the circles represent the proportion of events detected for Rekambo (in white) and other communities (in black) by each camera. Created with R v.4.0.2 (R Core Team 2020)

**Table S1 Camera-trap events of foreign chimpanzee communities**

| **Camera** | **Location** | **Date** | **Foreign chimpanzees** | |
| --- | --- | --- | --- | --- |
| 3 Sacco | Core area^1^ | 15/12/2018 | 2 AM, 1 JU, 2 UU | |
| CR | Out of range^3^ | 06/06/2017 | 1 AM, 1 AF, 1 JU, 1 IU | |
| CR | Out of range^3^ | 18/09/2018 | 1 AM, 1 ADM | |
| Dacryodes | Core area^1^ | 28/05/2018 | 1 AM, 1 JU | |
| Dacryodes | Core area^1^ | 16/09/2018 | 4 AM, 2 AU, 1 ADF, 1 JM, 1 JU | |
| Golden cat | Core area^2^ | 03/05/2018 | 1 AM | |
| Lara^P^ | Core area^2^ | 21/06/2017 | 8 AM, 2 AF, 1 ADM, 1 ADF, 2 JU | |
| Lara | Core area^2^ | 06/06/2018 | 1 AM, 1 AF, 1 ADU | |
| Lara^P^ | Core area^2^ | 06/09/2018 | 6 AM, 2 ADM, 1 AF, 1 IU | |
| Lulu^P^ | Core area^1^ | 20/9/2018 | 2 AM, 3 ADM | |
| Marc | Periphery^3^ | 13/08/2017 | 1 ADM | |
| Marc | Periphery^3^ | 16/12/2017 | 2 AM, 1 AU | |
| Marc | Periphery^3^ | 22/12/2017 | 3 AM, 2 AF, 1 ADM, 1 ADU, 4 UU | |
| Marc | Periphery^3^ | 23/12/2017 | 1 AF, 1 ADM, 1 IU | |
| Marc | Periphery^3^ | 28/12/2017 | 1 AF, 1 ADM, 1 IU | |
| Marcy | Periphery^3^ | 06/04/2018 | 1 AM, 1 AF, 1 ADF, 1 ADU, 1 IU | |
| Marcy | Periphery^3^ | 26/06/2018 | 1 AM, 2 AF, 1 ADF | |
| Marcy | Periphery^3^ | 25/09/2018 | 1 AM, 3 UU | |
| Monie | Core area^1^ | 17/08/2017 | 1 AF, 1 ADM | |
| Monie | Core area^1^ | 31/07/2018 | 1 ADM, 1 ADF | |
| Perroquet^P^ | Core area^1^ | 02/01/2019 | 6 AM, 3 AU, 1 ADM, 1 ADF, 2 ADU | |
| Rekambo1 | Periphery^3^ | 19/02/2019 | 1 AM, 1 ADU | |
| Rekambo2 | Periphery^3^ | 01/03/2018 | 2 AM, 2 AF, 1 ADF, 2 JU, 1 UU | |
| Rekambo2 | Periphery^3^ | 09/04/2018 | 1 AM, 2 AF, 1 IU | |
| Rekambo2 | Periphery^3^ | 10/12/2018 | 2 AM | |
| Rekambo3 | Periphery^2^ | 30/09/2017 | 1 AM, 1 AF, 1 JM | |
| Traverse^P^ | Core area^1^ | 01/06/2017 | 1 AM, 1 ADM, 1 ADF | |
| Traverse | Core area^1^ | 7/11/2017 | 6 AM, 6 AF, 2 AU, 1 ADM, 1 ADF, 2 JM, 1 IU | |
| Table S1. Shows the events of communities other than Rekambo recorded by the network of cameras (see Fig. S1) between May 2017-March 2019. If the event contained a patrol, the camera's name has the superscript P. Camera-trap events were considered to show patrols, when the chimpanzees recorded, were traveling silently and close to each other, wary, scanning the environment, sometimes sniffing ground and vegetation (Mitani and Watts 2005). Locations are label according to the Minimum Convex Polygon (MCP) home range as: Core area: within 75% MCP; Periphery: between 75-95% MCP; Out of range: outside 95% MCP. Locations according to the Biased Random Bridges (BRB) home range are labeled with the following superscripts: 1) Within 75% BRB; 2) 75-95% BRB; 3) Outside 95% BRB. Individuals were labeled as follows: Number of individuals + Age category + Sex. A=Adult, AD=Adolescent, F=Female, J=Juvenile, I= Infant, M= Male, and U=Unknown. | | | |  |

| 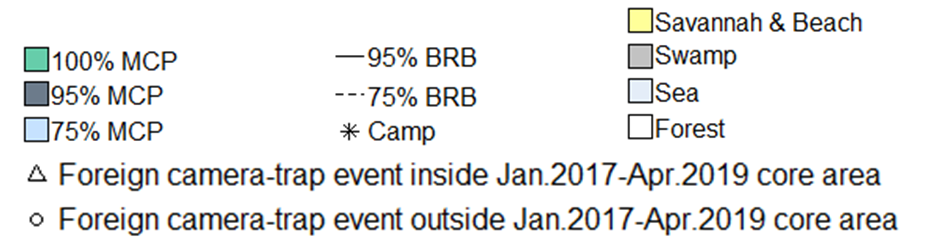 | |
| --- | --- |
| *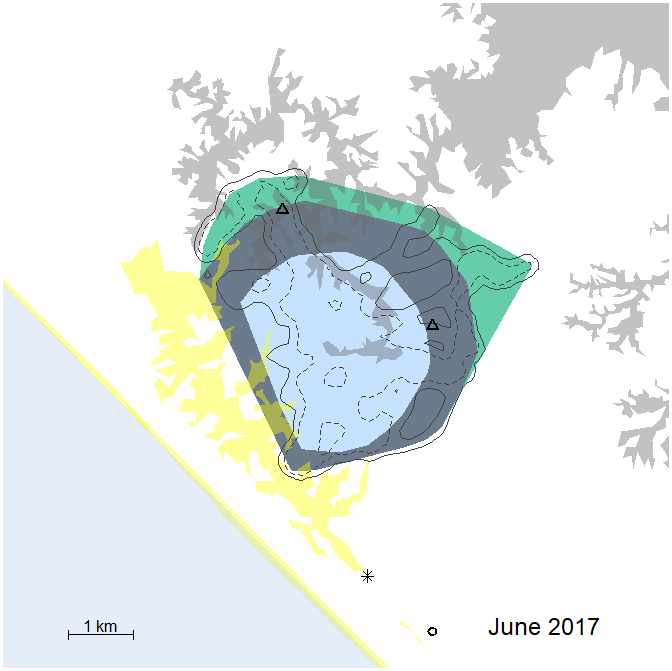* | *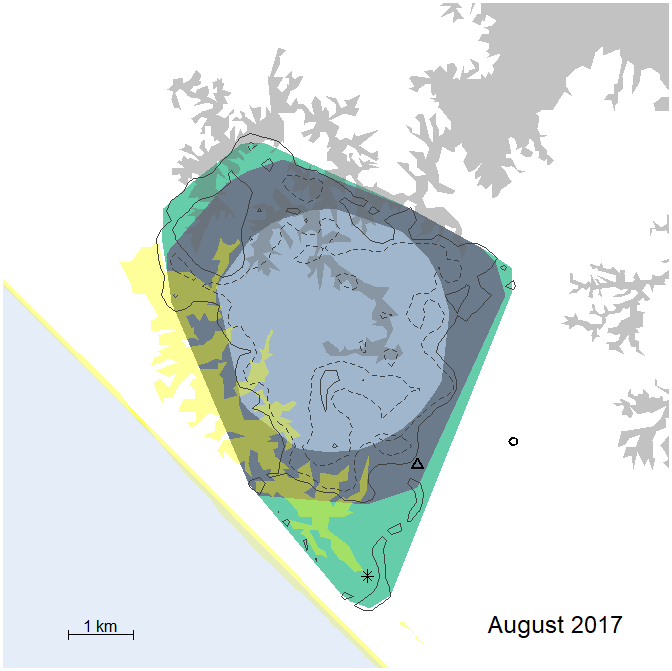* |
| *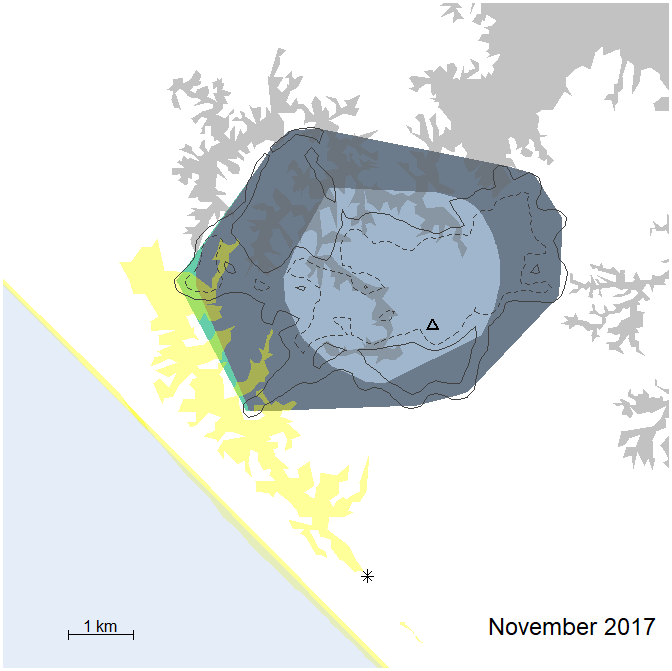* | 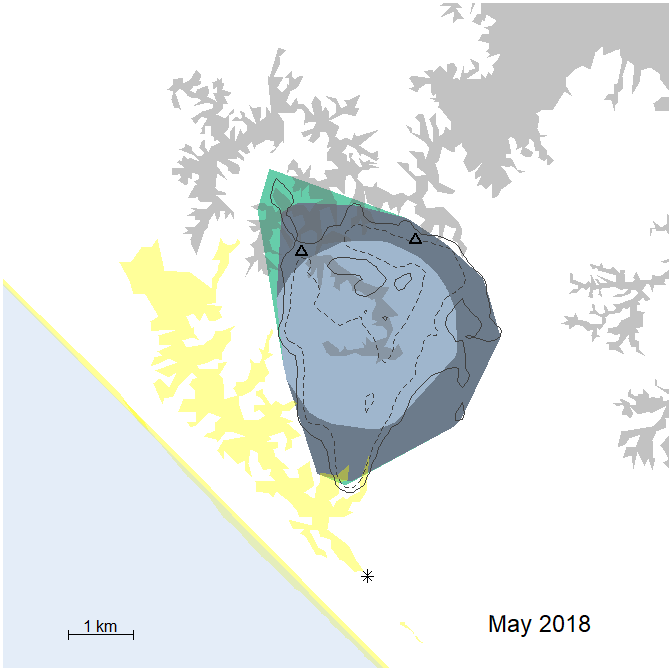 |
| **Fig. S3 Maps of Rekambo monthly home ranges in which foreign communities were recorded with the cumulative core areas (i.e., January 2017-April 2019) by the camera-trap network.** June 2017 (28 track-logs; 25,638 locations), August 2017 (31 track-logs; 31,703 locations), November 2017 (30 track-logs; 33,483 locations), May 2018 (20 track-logs; 32,415 locations), June 2018 (23 track-logs; 37,937 locations), July 2018 (18 track-logs; 16,537 locations), September 2018 (30 track-logs; 44,159 locations), December 2018 (29 track-logs; 30,413 locations), and January 2019 (30 track-logs; 34,503 locations). Created with R version R (v.4.0.2, R Core Team 2020) | |

| 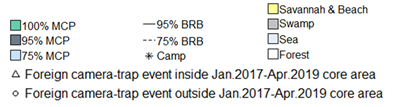 | |
| --- | --- |
| 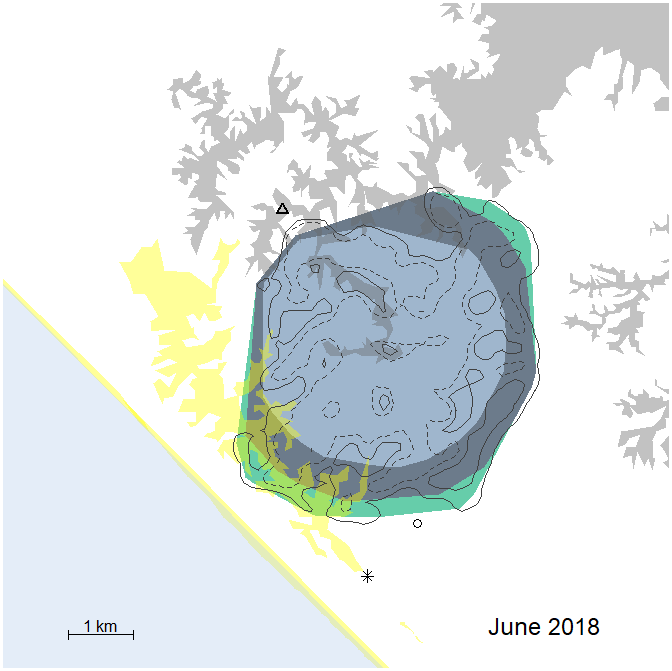 | *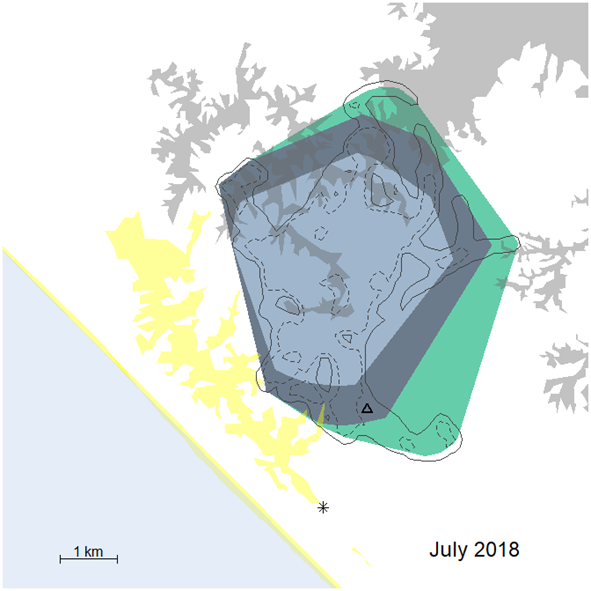* |
| *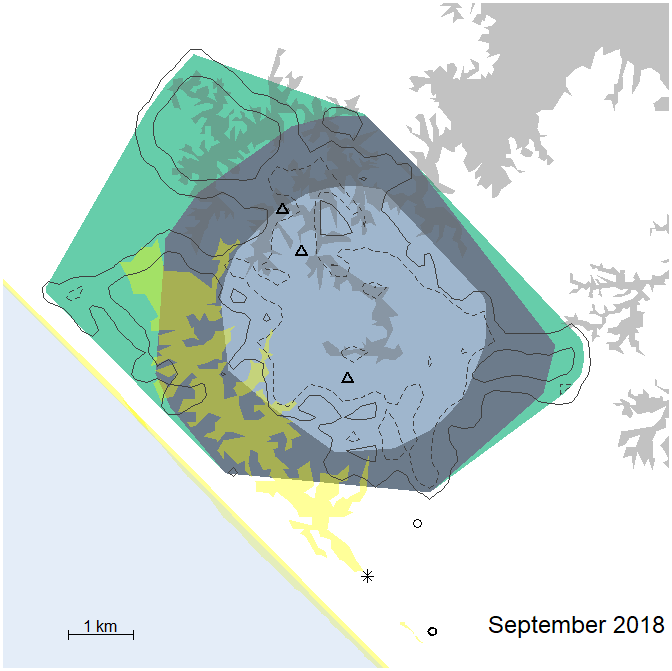* | 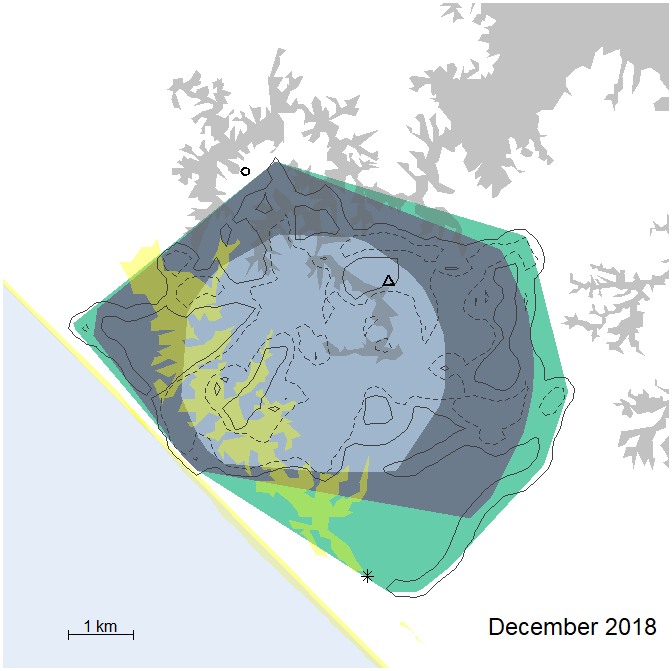 |
| **Fig. S3 (Cont.)** | |

| 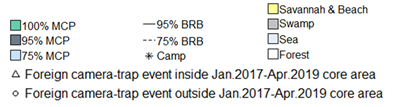 |
| --- |
| *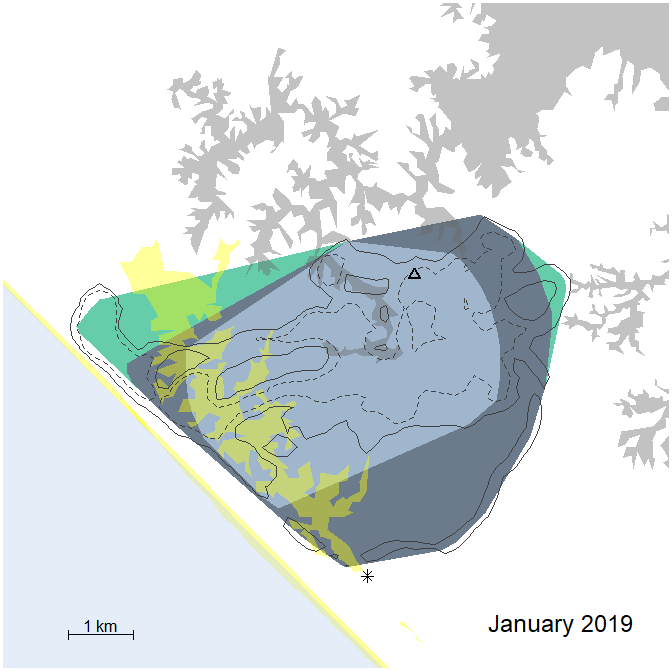* |
| **Fig. S3 (Cont. II)** |

|  |
| --- |
| 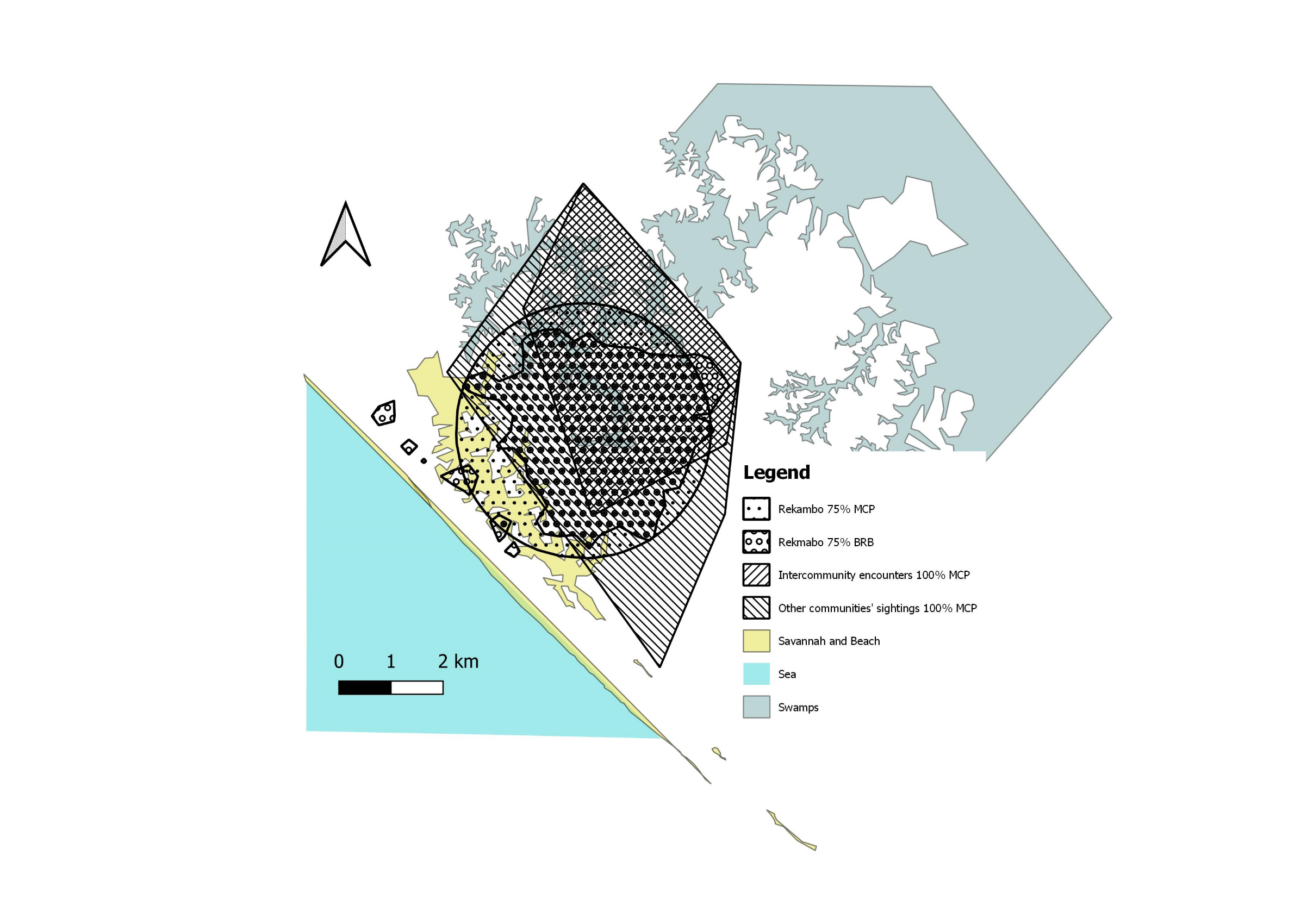 |

**Fig. S4 Overlap between 100% MCP created with sightings from other communities (i.e., camera-trap events plus encounters), 100% MCP created with intergroup encounter locations, and the core area (75% MCP and BRB, January 2017-April 2019) of the Rekambo community**. Polygons created with R version 4.0.0 (R Core Team 2020) and map with QGIS 3.16.1-Hannover, QGIS Development Team 2020).

# R code for maps and overlap estimations

Note: This code was designed to be run directly in R. Maps may be slightly different if the code is run in Rstudio (e.g., legends and scale may be placed incorrectly)

Table of Contents

[#### 1. Preparing R and the data for analysis 12](#_Toc64909500)

[# 1.1 Installing and activating all the needed packages 12](#_Toc64909501)

[# 1.2 Uploading the landscape shapes for the map 13](#_Toc64909502)

[# 1.3. Uploading the tracks 13](#_Toc64909503)

[# 1.4 Eliminating duplicates (sometimes it happened with some time stamps) and NA (problems with lag calculation) 13](#_Toc64909504)

[# 1.5 Preparing the coordinates for the calculations (from WGS to UTM and transformed into spatial points) 14](#_Toc64909505)

[# 1.6 Counting amount of data used in the calculations 14](#_Toc64909506)

[#### 2. Representing the camera-trap events, encounters and MCP home range 15](#_Toc64909507)

[# 2.1 Calculating the MCP (January 2017-April 2019) 15](#_Toc64909508)

[# 2.2 Uploading the camera-trap data 15](#_Toc64909509)

[# 2.3 Plotting the data over the map of the study site 16](#_Toc64909510)

[#### 3. Representing the camera-trap events, encounters and the home range as MCP and BRB 22](#_Toc64909511)

[# 3.1 Calculating the BRB 22](#_Toc64909512)

[# 3.2 Plotting the data over the map of the study site 22](#_Toc64909513)

[#### 4. Creating the shp to calculate in QGIS the overlap between the core area of Rekambo and the sightings of other communities 28](#_Toc64909514)

[# 4.1 Creating the shp files of the 75% MCP and 75%BRB (core areas, jan.2017-apr.2019)of the Rekambo community 28](#_Toc64909515)

[# 4.2 Creating the shp files of MCP created with sightings of other communities 28](#_Toc64909516)

[#### 5. Exploring whether there is temporal spatial partitioning between communities at the study site 29](#_Toc64909517)

[# 5.1 Looking at the sightings of other communities relative to the monthly home range of the Rekambo community in June 2017 30](#_Toc64909518)

[# 5.2 Looking at the sightings of other communities relative to the monthly home range of the Rekambo community in August 2017 34](#_Toc64909519)

[# 5.3 Looking at the sightings of other communities relative to the monthly home range of the Rekambo community in November 2017 39](#_Toc64909520)

[# 5.4 Looking at the sightings of other communities relative to the monthly home range of the Rekambo community in May 2018 43](#_Toc64909521)

[# 5.5 Looking at the sightings of other communities relative to the monthly home range of the Rekambo community in June 2018 48](#_Toc64909522)

[# 5.6 Looking at the sightings of other communities relative to the monthly home range of the Rekambo community in July 2018 52](#_Toc64909523)

[# 5.7 Looking at the sightings of other communities relative to the monthly home range of the Rekambo community in September 2018 57](#_Toc64909524)

[# 5.8 Looking at the sightings of other communities relative to the monthly home range of the Rekambo community in December 2018 61](#_Toc64909525)

[# 5.9 Looking at the sightings of other communities relative to the monthly home range of the Rekambo community in January 2019 66](#_Toc64909526)

#####################################################################

## #### 1. Preparing R and the data for analysis

#####################################################################

### # 1.1 Installing and activating all the needed packages

if(!require(installr)) {

install.packages("installr"); require(installr)} #load / install+load installr#Helps to automatically detect in a new version of R needs to be installed

# using the package:

updateR() # this will start the updating process of your R installation. It will check for newer versions, and if one is available, will guide you through the decisions you'd need to make.

install.packages("devtools")#package needed to automatically check if it is necessary to install Rtools

library(devtools)

#REMEMBER TO INSTAL THE PROPER VERSION OF RTOOLS FROM: https://cran.r-project.org/bin/windows/Rtools/

install.packages("shapefiles") #Needed to import the shapes of the savannah, swamps, beach and sea

install.packages("rgdal")

install.packages("adehabitatHR") #Needed to calculate MCP and BRB

install.packages("splancs")# Needed to use the object areapl to calculate the different MCP contours

install.packages("adehabitatLT")#Needed to transform the tracklog data into a ltraj object so they can be used to calculate BBMM and BRB in adehabitatHR

install.packages("caTools")

install.packages("bitops")

install.packages("raster")

install.packages("adehabitatMA")

install.packages("maptools")

library(shapefiles)

library(rgdal)

library(adehabitatHR)

library(splancs)

library(adehabitatLT)

library(caTools)

library(bitops)

library(raster)

library(adehabitatMA)

library(maptools)

### # 1.2 Uploading the landscape shapes for the map

source("C:/Users/laura/OneDrive/Research/LoangoChimpanzeeProject/201909_intercommunity_study/R_map/split_shape.r")

source("C:/Users/laura/OneDrive/Research/LoangoChimpanzeeProject/201909_intercommunity_study/R_map/geo_functions_bcgps.r")

savannah=read.shp("C:/Users/laura/OneDrive/Research/LoangoChimpanzeeProject/201909_intercommunity_study/R_map/Savannah_polygone.shp")$shp

savannah=split.shape(shp=savannah)

swamp=read.shp("C:/Users/laura/OneDrive/Research/LoangoChimpanzeeProject/201909_intercommunity_study/R_map/swamps1.shp")$shp

swamp=split.shape(shp=swamp)

sea=read.shp("C:/Users/laura/OneDrive/Research/LoangoChimpanzeeProject/201909_intercommunity_study/R_map/Sea.shp")$shp

sea=split.shape(shp=sea)

### # 1.3. Uploading the tracks

alltracks=read.table(file="C:/Users/laura/OneDrive/Research/LoangoChimpanzeeProject/201909_intercommunity_study/2019refresh/202004_maps/202004_tracklogs_201701_to_201904.txt",sep="\t", header=TRUE)

### # 1.4 Eliminating duplicates (sometimes it happened with some time stamps) and NA (problems with lag calculation)

dup.ts=as.data.frame(duplicated(alltracks$date))#Like this we can see which rows have a duplicated time stamp in case we want to dig deeper

summary(dup.ts)#75623 duplicated time stamps

anyDuplicated(alltracks$date)

alltracks=alltracks[!duplicated(alltracks$date), ]#Eliminate rows with duplicated time stamp

anyDuplicated(alltracks$date)

any(is.na(alltracks))

alltracks<-na.omit(alltracks)

apply(alltracks, 2, function(x) any(is.infinite(x)))#No infinite values detected

### # 1.5 Preparing the coordinates for the calculations (from WGS to UTM and transformed into spatial points)

utm.coords=WGS.to.UTM(long=alltracks$lon, lat=alltracks$lat, utm.zone=utm.zone(mean(alltracks$lon), mean(alltracks$lat)), avoid.negatives=T, return.zone=F)

tracks=utm.coords

xyALL=SpatialPoints(coords=tracks[, c("long", "lat")])

### # 1.6 Counting amount of data used in the calculations

alltracks$yyyymmdd<-as.factor(alltracks$yyyymmdd)

alltracks$burst<-as.factor(alltracks$burst)

str(alltracks)

#No obs=No locations= 724286

#$burst levels=No tracks = 690

#$yyyymmdd= No. obs. days=644

#Calculating observation time included in the sample

#We will calculate the observation time with the calculation on lag. We will need to remove any with a

#lag >30min, which would be joining different tracks, so time in which there was no chimpanzee observation

lags<- alltracks[alltracks$lag <= 30,]

lags<- lags$lag/60

str(lags)

sum(lags)#6153h approx

#####################################################################

## #### 2. Representing the camera-trap events, encounters and MCP home range

#####################################################################

setwd("C:/Users/laura/OneDrive/Research/LoangoChimpanzeeProject/201909_intercommunity_study/2019refresh/202004_maps/")

### # 2.1 Calculating the MCP (January 2017-April 2019)

to.get=c(100, 95, 75)

MCP=vector("list", length(to.get))

names(MCP)=to.get

for(i in 1:length(to.get)){

xxALL=mcp(xyALL, percent=to.get[i])

MCP[[i]]=xxALL@polygons[[1]]@Polygons[[1]]@coords

}

MCP.areas=unlist(lapply(MCP, areapl))

MCP.areas=MCP.areas/(1000^2)

names(MCP.areas)=to.get

MCP.areas

### # 2.2 Uploading the camera-trap data

cam.loc.data=read.table(file="C:/Users/laura/OneDrive/Research/LoangoChimpanzeeProject/201909_intercommunity_study/2019refresh/201908_map/201909_camera_trap_data_map.txt", header=T, sep="\t")

cam.loc.data$Correction=cam.loc.data$Correction/max(cam.loc.data$Correction, na.rm=T)

### # 2.3 Plotting the data over the map of the study site

#### ## 2.3.1 Plotting the MCP and the landscape features

par(mar=rep(0.2, 4))

plot(as.matrix(MCP[["100"]]), asp=1, xlab="", ylab="", axes=T, type="n", xaxt="n", yaxt="n",

ylim=range(c(cam.loc.data$y, as.matrix(MCP[["100"]])[, "lat"])), bty="n")

cols=c("gray45", "gray65", "gray80")

for(i in 1:length(to.get)){

polygon(MCP[[i]], border=NA, col=cols[i])

}

for(i in 1:length(savannah)){

polygon(savannah[[i]], border=1, col="black", density=20, angle=15)

}

polygon(swamp[[10]],border=1 , col="black", density=40, angle=90)

polygon(sea[[1]], border=1 , col="black", density=20, angle=0, lty=3)

#### ## 2.3.2 Adding camera-traps

source("C:/Users/laura/OneDrive/Research/LoangoChimpanzeeProject/201909_intercommunity_study/2019refresh/202004_maps/Rscripts_RM/pie_2_poly.r")

##Pie chart function

make.pie<-function(xarea, fract, resol=100){

xnames=names(fract)

if(sum(fract>0)){

fract=c(0, cumsum(fract/sum(fract)))*2*pi

radius=sqrt(xarea/pi)

#browser()

ires=lapply(1:(length(fract)-1), function(x){

rads=seq(fract[x], fract[x+1], length.out=resol)

return(list(x=radius*c(0, sin(rads), 0), y=radius*c(0, cos(rads), 0)))

})

#names(ires)=xnames

}else{

ires=NULL

}

circ=seq(0, 2*pi, length.out=resol)

circ=list(x=radius*sin(circ), y=radius*cos(circ))

return(list(polies=ires, circ=circ)) }}

##Representing the camera-trap data on pie charts over the map

pie.cols=grey(level=c(0, 1))

for(i in 1:nrow(cam.loc.data)){

if(!is.na(cam.loc.data$Correction[i])){

if(cam.loc.data$No_videos[i]==0){

xpie=make.pie(xarea=cam.loc.data$Correction[i], fract=as.matrix(cam.loc.data[i, rev(c("number_view_rekambo", "nb_view_foreign"))]))

#browser()

if(cam.loc.data[i, "nb_view_foreign"]>0){

for(j in 1:length(xpie$polies)){

polygon(x=cam.loc.data$x[i]+xpie$polies[[j]]$x*500, y=cam.loc.data$y[i]+xpie$polies[[j]]$y*500, col=pie.cols[j])

}

}else{

len=length(xpie[[2]]$x)

polygon(x=cam.loc.data$x[i]+xpie$polies[[2]]$x[2:(len-1)]*500, y=cam.loc.data$y[i]+xpie$polies[[2]]$y[2:(len-1)]*500, col=pie.cols[2])

}

}else{

xpie=make.pie(xarea=cam.loc.data$Correction[i], fract=c(1, 0))

len=length(xpie[[2]]$x)

polygon(x=cam.loc.data$x[i]+xpie$polies[[1]]$x[2:(len-1)]*500, y=cam.loc.data$y[i]+xpie$polies[[1]]$y[2:(len-1)]*500, col="white")

}

}

}

#### ## 2.3.3 Adding the locations of intercommunity encounters

#adding acoustic encounter points:

xdata1=read.table(file="C:/Users/laura/OneDrive/Research/LoangoChimpanzeeProject/201909_intercommunity_study/202006_rekambo_intercommunity_study/PeerReview/202101_acoustic_encounters.txt", header=T, sep="\t")

for(i in 1:nrow(xdata1)){

points(x=xdata1$x[i], y=xdata1$y[i], pch=65, cex=1.5)}

#adding visual encounters:

xdata2=read.csv(file="C:/Users/laura/OneDrive/Research/LoangoChimpanzeeProject/201909_intercommunity_study/202006_rekambo_intercommunity_study/PeerReview/202101_visualencounters.txt", header=T, sep="\t")

for(i in 1:nrow(xdata2)){

points(x=xdata2$x[i], y=xdata2$y[i], pch=86, cex=1.5)}

#adding physical encounter:

xdata3=read.csv(file="C:/Users/laura/OneDrive/Research/LoangoChimpanzeeProject/201909_intercommunity_study/202006_rekambo_intercommunity_study/PeerReview/202101_physicalencounter.txt", header=T, sep="\t")

for(i in 1:nrow(xdata3)){

points(x=xdata3$x[i], y=xdata3$y[i], pch=80, cex=1.5)}

#add intercommunity killings (2017-2019):

xdata4=read.csv(file="C:/Users/laura/OneDrive/Research/LoangoChimpanzeeProject/201909_intercommunity_study/202006_rekambo_intercommunity_study/PeerReview/202101_intercommunitykillings.txt", header=T, sep="\t")

for(i in 1:nrow(xdata4)){

points(x=xdata4$x[i], y=xdata4$y[i], pch=75, cex=1.5)}

#adding infanticides of unknown community (intra o inter community?):

xdata5=read.csv(file="C:/Users/laura/OneDrive/Research/LoangoChimpanzeeProject/201909_intercommunity_study/202006_rekambo_intercommunity_study/PeerReview/202101_infanticides_unknowncommunity.txt", header=T, sep="\t")

for(i in 1:nrow(xdata5)){

points(x=xdata5$x[i], y=xdata5$y[i], pch=85, cex=1.5)}

#add killings before 2017:

xdata6=read.csv(file="C:/Users/laura/OneDrive/Research/LoangoChimpanzeeProject/201909_intercommunity_study/2019refresh/201908_killings_before_2017.txt", header=T, sep="\t")

#polygons for stars:

nodes=seq(from=0, to=2*pi, length.out=11)+pi/2

mult=sqrt((cos(pi/2-2*pi/10)*sin(pi/2-2*pi/5)/sin(pi/2-2*pi/10))^2+sin(pi/2-2*pi/5)^2)

mult=c(rep(c(1, mult), times=5), 1)

poly2=cbind(x=cos(nodes)*mult*100, y=sin(nodes)*mult*100)

for(i in 1:nrow(xdata6)){

poly3=poly2

poly3[, "x"]=poly3[, "x"]+xdata6$x[i]

poly3[, "y"]=poly3[, "y"]+xdata6$y[i]

polygon(poly3, border="black", col="black")

}

#### ## 2.3.4 Adding the location of the Ozouga Research Camp to the map

camp.loc=c(x=553601, y=9765888)

points(x=camp.loc["x"], y=camp.loc["y"], pch=8, cex=1.2, lwd=1)

#### ## 2.3.5 Adding the scale

#### arrows(x0=par()[["usr"]][1]+8000, x1=par()[["usr"]][1]+9000, y0=par()[["usr"]][3]+500, y1=par()[["usr"]][3]+500, code=3, angle=90, length=0.05)

#### text(x=par()[["usr"]][1]+8500, y=par()[["usr"]][3]+700, labels="1 km",cex=1.5)

#### ## 2.3.6. Saving the map

savePlot(file="C:/Users/laura/OneDrive/Research/LoangoChimpanzeeProject/201909_intercommunity_study/202006_rekambo_intercommunity_study/PeerReview/202101_map_intercommunity_study_MCP100_95_75.png", type="png")

dev.copy2pdf(file="C:/Users/laura/OneDrive/Research/LoangoChimpanzeeProject/201909_intercommunity_study/202006_rekambo_intercommunity_study/PeerReview/202104_intercommunity_MCP100_95_75.pdf")

#### ## 2.3.7 Creating the legend

par(mar=rep(0.2, 4))

plot(as.matrix(MCP[["100"]]), asp=1, xlab="", ylab="", axes=T, type="n", xaxt="n", yaxt="n",

ylim=range(c(cam.loc.data$y, as.matrix(MCP[["100"]])[, "lat"])), bty="n")

box.el=300

texts=c(paste(c(100, 95, 75), "% MCP", sep=""), "Savannah & Beach", "Swamp", "Sea","Forest",

"Acoustic Encounter", "Visual Encounter", "Physical Encounter", "Intercommunity Killing (2017-2019)", "Unknown Community Killing (2017-2019)","Killing (before 2017)",”Rekambo camera-trap event", "Other communities' camera-trap event", "Camp")

cols=c("gray45", "gray65", "gray80", NA, NA, NA, "white",NA,NA,NA,NA,NA,"black", "white", "black", "black")

borders=rep("black", length(cols))

densities =c(rep(NA, 3), 20, 40,20,rep(NA, 10))

angles =c(rep(NA, 3), 15, 90,0,rep(NA, 10))

pchs=c(rep(NA, 7), 65,86, 80, 75,85, rep(NA,4))

ltys=c(rep(1, 5), 3,rep(1,10))

polies=vector("list", length(texts))

size.fac=0.8

polies[[13]]=poly2

circ=seq(0, 2*pi, length.out=500)

size.fac=80

polies[[14]]=cbind(x=size.fac*sin(circ), y=size.fac*cos(circ))

polies[[15]]=cbind(x=size.fac*sin(circ), y=size.fac*cos(circ))

for(i in 1:length(texts)){

if(i<=7){

rect(xleft=par()[["usr"]][1]+0.5*box.el, xright=par()[["usr"]][1]+1.5*box.el,

ytop= par()[["usr"]][4]-box.el*0.5*i-box.el*(i-1),

ybottom=par()[["usr"]][4]-box.el*0.5*i-box.el*(i-1)-box.el,

col=cols[i], border= "black", density=densities[i], angle=angles[i],lty=ltys[i])

}else if(i<=12){

points(x=par()[["usr"]][1]+box.el, y=par()[["usr"]][4]-box.el*0.5*i-box.el*(i-1)-box.el/2, pch=pchs[i])

}else if(i<=15){

polygon(x=par()[["usr"]][1]+box.el+polies[[i]][, 1], y=par()[["usr"]][4]-box.el*0.5*i-box.el*(i-1)-box.el/2+polies[[i]][, 2], col=cols[i], border=borders[i])

}else{

points(x=par()[["usr"]][1]+box.el, y=par()[["usr"]][4]-box.el*0.5*i-box.el*(i-1)-box.el/2, pch=8)

}

text(x=par()[["usr"]][1]+1.5*box.el+50, y=par()[["usr"]][4]-box.el*0.5*i-box.el*(i-1)-box.el/2, labels=texts[i], adj=0, cex=1.0)

}

#### ## 2.3.8 Saving the legend

savePlot(file="C:/Users/laura/OneDrive/Research/LoangoChimpanzeeProject/201909_intercommunity_study/202006_rekambo_intercommunity_study/PeerReview/202101_legend_intercommunity_study_MCP100_95_75.png", type="png")

dev.copy2pdf(file="C:/Users/laura/OneDrive/Research/LoangoChimpanzeeProject/201909_intercommunity_study/202006_rekambo_intercommunity_study/PeerReview/202101_legend_intercommunity_MCP100_95_75.pdf")

#####################################################################

## #### 3. Representing the camera-trap events, encounters and the home range as MCP and BRB

#####################################################################

# This codes assumes that the point 2 was fully run before running 3

### # 3.1 Calculating the BRB

# Transforming the date into POSIXct format to create the ltraj object to calculate BRB

date.brb <- as.POSIXct(strptime(alltracks$date,format="%d-%m-%Y %H:%M:%S"))

#Creating ltraj object

ltraj=as.ltraj(xy = tracks, date = date.brb, id=F)

#Calculating the BRB

rek.brb <- BRB(ltraj, D = 1.6,, Tmax = 120*60, Lmin = 50, hmin=100,type="UD", grid = 100, extent=1)

### # 3.2 Plotting the data over the map of the study site

#### # 3.2.1 Plotting the MCP and the landscape features

par(mar=rep(0.2, 4))

plot(as.matrix(MCP[["100"]]), asp=1, xlab="", ylab="", axes=T, type="n", xaxt="n", yaxt="n",

ylim=range(c(cam.loc.data$y, as.matrix(MCP[["100"]])[, "lat"])), bty="n")

cols=c("gray45", "gray65", "gray80")

for(i in 1:length(to.get)){

polygon(MCP[[i]], border=NA, col=cols[i])

}

for(i in 1:length(savannah)){

polygon(savannah[[i]], border=1, col="black", density=20, angle=15)

}

polygon(swamp[[10]],border=1 , col="black", density=40, angle=90)

polygon(sea[[1]], border=1 , col="black", density=20, angle=0, lty=3)

}

#### # 3.2.2 Adding the BRB

brb.cont=rev(c( 75, 95))

brb.areas=rep(0, length(brb.cont))

names(brb.areas)=brb.cont

ltys=c(1,3)

for(k in 1:length(brb.cont)){

ver.brb=getverticeshr(rek.brb, percent=brb.cont[k])

for(i in 1:length(ver.brb@polygons[[1]]@Polygons)){

polygon(ver.brb@polygons[[1]]@Polygons[[i]]@coords, border=grey(level=0.25), col=NA, lty=ltys[k], lwd=3)

brb.areas[k]=brb.areas[k]+areapl(ver.brb@polygons[[1]]@Polygons[[i]]@coords)/(1000^2)

}

}

brb.areas# These are the BRB areas in km2

#### # 3.3.3 Adding camera-traps

pie.cols=grey(level=c(0, 1))

for(i in 1:nrow(cam.loc.data)){

if(!is.na(cam.loc.data$Correction[i])){

if(cam.loc.data$No_videos[i]==0){

xpie=make.pie(xarea=cam.loc.data$Correction[i], fract=as.matrix(cam.loc.data[i, rev(c("number_view_rekambo", "nb_view_foreign"))]))

#browser()

if(cam.loc.data[i, "nb_view_foreign"]>0){

for(j in 1:length(xpie$polies)){

polygon(x=cam.loc.data$x[i]+xpie$polies[[j]]$x*500, y=cam.loc.data$y[i]+xpie$polies[[j]]$y*500, col=pie.cols[j])

}

}else{

len=length(xpie[[2]]$x)

polygon(x=cam.loc.data$x[i]+xpie$polies[[2]]$x[2:(len-1)]*500, y=cam.loc.data$y[i]+xpie$polies[[2]]$y[2:(len-1)]*500, col=pie.cols[2])

}

}else{

xpie=make.pie(xarea=cam.loc.data$Correction[i], fract=c(1, 0))

len=length(xpie[[2]]$x)

polygon(x=cam.loc.data$x[i]+xpie$polies[[1]]$x[2:(len-1)]*500, y=cam.loc.data$y[i]+xpie$polies[[1]]$y[2:(len-1)]*500, col="white")

}

}

}

#### # 3.3.4 Adding the locations of the intercommunity encounters

#adding acoustic encounter points:

for(i in 1:nrow(xdata1)){

points(x=xdata1$x[i], y=xdata1$y[i], pch=65, cex=1.5)}

#adding visual encounters:

for(i in 1:nrow(xdata2)){

points(x=xdata2$x[i], y=xdata2$y[i], pch=86, cex=1.5)}

#adding physical encounter:

for(i in 1:nrow(xdata3)){

points(x=xdata3$x[i], y=xdata3$y[i], pch=80, cex=1.5)}

#add intercommunity killings (2017-2019):

for(i in 1:nrow(xdata4)){

points(x=xdata4$x[i], y=xdata4$y[i], pch=75, cex=1.5)}

#adding infanticides of unknown community (intra o inter community?):

for(i in 1:nrow(xdata5)){

points(x=xdata5$x[i], y=xdata5$y[i], pch=85, cex=1.5)}

#add killings before 2017:

#polygons for stars:

nodes=seq(from=0, to=2*pi, length.out=11)+pi/2

mult=sqrt((cos(pi/2-2*pi/10)*sin(pi/2-2*pi/5)/sin(pi/2-2*pi/10))^2+sin(pi/2-2*pi/5)^2)

mult=c(rep(c(1, mult), times=5), 1)

poly2=cbind(x=cos(nodes)*mult*100, y=sin(nodes)*mult*100)

for(i in 1:nrow(xdata6)){

poly3=poly2

poly3[, "x"]=poly3[, "x"]+xdata6$x[i]

poly3[, "y"]=poly3[, "y"]+xdata6$y[i]

polygon(poly3, border="black", col="black")

}

#### ## 3.3.5 Adding the location of the Ozouga Research Camp to the map

camp.loc=c(x=553601, y=9765888)

points(x=camp.loc["x"], y=camp.loc["y"], pch=8, cex=1.2, lwd=1)

#### ## 3.3.6 Adding the scale

arrows(x0=par()[["usr"]][1]+8000, x1=par()[["usr"]][1]+9000, y0=par()[["usr"]][3]+500, y1=par()[["usr"]][3]+500, code=3, angle=90, length=0.05)

text(x=par()[["usr"]][1]+8500, y=par()[["usr"]][3]+700, labels="1 km",cex=1.5)

#### ## 3.3.7 Saving the map

savePlot(file="C:/Users/laura/OneDrive/Research/LoangoChimpanzeeProject/201909_intercommunity_study/202006_rekambo_intercommunity_study/PeerReview/202101_map_intercommunity_study_MCP_BRB.png", type="png")

dev.copy2pdf(file="C:/Users/laura/OneDrive/Research/LoangoChimpanzeeProject/201909_intercommunity_study/202006_rekambo_intercommunity_study/PeerReview/2202101_intercommunity_MCP100_BRB.pdf")

#### ## 3.3.8 Creating the legend

par(mar=rep(0.2, 4))

plot(as.matrix(MCP[["100"]]), asp=1, xlab="", ylab="", axes=T, type="n", xaxt="n", yaxt="n",

ylim=range(c(cam.loc.data$y, as.matrix(MCP[["100"]])[, "lat"])), bty="n")

box.el=300

texts=c(paste(c(100, 95, 75), "% MCP", sep=""), "Savannah & Beach", "Swamp", "Sea","Forest", "BRB 95%", "BRB 75%",

"Acoustic Encounter", "Visual Encounter", "Physical Encounter", "Intercommunity Killing (2017-2019)", "Unknown Community Killing (2017-2019)","Killing (before 2017)",,"Killing (before 2017)","Rekambo camera-trap event", "Other communities' camera-trap event", "Camp")

cols=c("gray45", "gray65", "gray80", NA, NA, NA, "white",NA,NA,NA,NA,NA,NA,NA, "black","white", "black", "black")

borders=rep("black", length(cols))

densities =c(rep(NA, 3), 20, 40,20,rep(NA, 12))

angles =c(rep(NA, 3), 15, 90,0,rep(NA, 12))

pchs=c(rep(NA, 7), 65,86, 80, 75,85, rep(NA,4))

ltys=c(rep(1, 5), 3,1,1,3, rep(1,10))

polies=vector("list", length(texts))

size.fac=0.8

polies[[15]]=poly2

circ=seq(0, 2*pi, length.out=500)

size.fac=80

polies[[16]]=cbind(x=size.fac*sin(circ), y=size.fac*cos(circ))

polies[[17]]=cbind(x=size.fac*sin(circ), y=size.fac*cos(circ))

for(i in 1:length(texts)){

if(i<=7){

rect(xleft=par()[["usr"]][1]+0.5*box.el, xright=par()[["usr"]][1]+1.5*box.el,

ytop= par()[["usr"]][4]-box.el*0.5*i-box.el*(i-1),

ybottom=par()[["usr"]][4]-box.el*0.5*i-box.el*(i-1)-box.el,

col=cols[i], border= "black", density=densities[i], angle=angles[i],lty=ltys[i])

}else if(i<=9){

segments(x0=par()[["usr"]][1]+0.5*box.el, x1=par()[["usr"]][1]+1.5*box.el,

y0=par()[["usr"]][4]-box.el*0.5*i-box.el*(i-1)-box.el/2, y1=par()[["usr"]][4]-box.el*0.5*i-box.el*(i-1)-box.el/2, lty=ltys [i], lwd=3)

}else if(i<=14){

points(x=par()[["usr"]][1]+box.el, y=par()[["usr"]][4]-box.el*0.5*i-box.el*(i-1)-box.el/2, pch=pchs[i])

}else if(i<=17){

polygon(x=par()[["usr"]][1]+box.el+polies[[i]][, 1], y=par()[["usr"]][4]-box.el*0.5*i-box.el*(i-1)-box.el/2+polies[[i]][, 2], col=cols[i], border=borders[i])

}else{

points(x=par()[["usr"]][1]+box.el, y=par()[["usr"]][4]-box.el*0.5*i-box.el*(i-1)-box.el/2, pch=8)

}

text(x=par()[["usr"]][1]+1.5*box.el+50, y=par()[["usr"]][4]-box.el*0.5*i-box.el*(i-1)-box.el/2, labels=texts[i], adj=0, cex=1.0)

}

#### ## 3.3.9 Saving the legend

savePlot(file="C:/Users/laura/OneDrive/Research/LoangoChimpanzeeProject/201909_intercommunity_study/202006_rekambo_intercommunity_study/PeerReview/202101_legend_intercommunity_study_MCP_BRB.png", type="png")

dev.copy2pdf(file="C:/Users/laura/OneDrive/Research/LoangoChimpanzeeProject/201909_intercommunity_study/202006_rekambo_intercommunity_study/PeerReview/202101_legend_intercommunity_MCP_BRB.pdf")

#####################################################################

## #### 4. Creating the shp to calculate in QGIS the overlap between the core area of Rekambo and the sightings of other communities

#####################################################################

setwd("C:/Users/laura/OneDrive/Research/LoangoChimpanzeeProject/201909_intercommunity_study/202006_rekambo_intercommunity_study/PeerReview/")

####

### # 4.1 Creating the shp files of the 75% MCP and 75%BRB (core areas, jan.2017-apr.2019)of the Rekambo community

####

MCP75=mcp(xyALL, percent=75)

writeOGR(MCP75, dsn="MCP75", driver = "ESRI Shapefile", layer="MCP75")

BRB75=getverticeshr(rek.brb, percent=75)

writeOGR(BRB75, dsn="BRB75", driver = "ESRI Shapefile", layer="BRB75")

####

### # 4.2 Creating the shp files of MCP created with sightings of other communities

####

#Uploading the sightings of other communities accounting for camera-trap sightings and intercommunity encounters

all_loc_ot=read.table(file="C:/Users/laura/OneDrive/Research/LoangoChimpanzeeProject/201909_intercommunity_study/202006_rekambo_intercommunity_study/PeerReview/202102_other_community_sightings_camera_traps_and_IE.txt", header=T, sep="\t")

#Uploading the sightings of other communities accounting for camera-trap sightings and intercommunity encounters

loc_ie=read.table(file="C:/Users/laura/OneDrive/Research/LoangoChimpanzeeProject/201909_intercommunity_study/202006_rekambo_intercommunity_study/PeerReview/202102_IEs_for_overlap_with_other_communities.txt", header=T, sep="\t")

#Converting the location of the sightings into SpatialPoints

xy_allOT=SpatialPoints(coords=all_loc_ot[, c("x", "y")])

xy_IE=SpatialPoints(coords=loc_ie[, c("x", "y")])

#Building MCPs with the sightings

MCP_allOT=mcp(xy_allOT, percent=100)

MCP_IE=mcp(xy_IE, percent=100)

#Creating shp for QGIS with the MCPs of other communities

writeOGR(MCP_allOT, dsn="MCP_allOT", driver = "ESRI Shapefile", layer="MCP_allOT")

writeOGR(MCP_IE, dsn="MCP_IE", driver = "ESRI Shapefile", layer="MCP_IE")

#####################################################################

## #### 5. Exploring whether there is temporal spatial partitioning between communities at the study site

#####################################################################

#This code assumes that the sightings of the other communities have already been uploaded in the previous analyses (4.Creating the shp to calculate in QGIS [...] )

#Splitting the travelling routes of the Rekambo community by year

tracks.2017<- alltracks[alltracks$year=="2017",]

tracks.2018 <- alltracks[alltracks$year =="2018",]

tracks.2019 <- alltracks[alltracks$year =="2019",]

####

### # 5.1 Looking at the sightings of other communities relative to the monthly home range of the Rekambo community in June 2017

####

#Preparing the data to calculate the Home Range of the Rekambo community in June 2017

tracks201706<- tracks.2017[tracks.2017$month=="6",]

utm.coords.201706=WGS.to.UTM(long=tracks201706$lon, lat=tracks201706$lat, utm.zone=utm.zone(mean(tracks201706$lon), mean(tracks201706$lat)), avoid.negatives=T, return.zone=F)

tracks.201706=utm.coords.201706

xy201706=SpatialPoints(coords=tracks.201706[, c("long", "lat")])

#Counting amount of data used in the calculations

tracks201706$burst<-as.factor(tracks201706$burst)

str(tracks201706)#burst=tracklog=28; relocations=25 638

##Calculating MCP

to.get=c(100, 95, 75)

MCP201706=vector("list", length(to.get))

names(MCP201706)=to.get

for(i in 1:length(to.get)){

xx201706=mcp(xy201706, percent=to.get[i])

MCP201706[[i]]=xx201706@polygons[[1]]@Polygons[[1]]@coords

}

MCP201706.areas=unlist(lapply(MCP201706, areapl))

MCP201706.areas=MCP201706.areas/(1000^2)

MCP201706.areas

#BRB of June 2017

#Date for ltraj needs to be in POSIXct format, so we have to transform it

date.brb.201706 <- as.POSIXct(strptime(tracks201706$date,format="%d-%m-%Y %H:%M:%S"))

#Create ltraj object

ltraj.201706=as.ltraj(xy = tracks.201706, date = date.brb.201706, id=F)

#Calculating BRB of June 2017

rek.brb.201706 <- BRB(ltraj.201706, D = 1.6, Tmax = 120*60, Lmin = 50, hmin=100,type="UD", grid = 100, extent=1)

#Mapping

par(mar=rep(0.2, 4))

plot(as.matrix(MCP201706[["100"]]), asp=1, xlab="", ylab="", axes=T, type="n", xaxt="n", yaxt="n",

xlim=c(548350, 557873),ylim=c(9765030, 9774195), bty="n")

cols=c("mediumaquamarine", "slategray4", "slategray1")

for(i in 1:length(to.get)){

polygon(MCP201706[[i]], border=NA, col=cols[i])

}

for(i in 1:length(savannah)){

polygon(savannah[[i]], border=NA, col=adjustcolor(col="yellow", alpha.f=0.4))

}

polygon(swamp[[10]], border=NA, col=grey(level=0.4, alpha=0.4))

polygon(sea[[1]], border=NA, col=adjustcolor(col="lightsteelblue2", alpha.f=0.4))

#adding brb:

brb201706.cont=rev(c(75, 95))

brb201706.areas=rep(0, length(brb201706.cont))

names(brb201706.areas)=brb201706.cont

for(k in 1:length(brb201706.cont)){

ver.brb201706=getverticeshr(rek.brb.201706, percent=brb201706.cont[k])

for(i in 1:length(ver.brb201706@polygons[[1]]@Polygons)){

polygon(ver.brb201706@polygons[[1]]@Polygons[[i]]@coords, border=grey(level=0.25), col=NA, lty=k)

brb201706.areas[k]=brb201706.areas[k]+areapl(ver.brb201706@polygons[[1]]@Polygons[[i]]@coords)/(1000^2)

}

}

brb201706.areas

#Adding the location of the Ozouga Research Camp

camp.loc=c(x=553601, y=9765888)

points(x=camp.loc["x"], y=camp.loc["y"], pch=8, cex=1.2, lwd=1)

#Adding the scale

arrows(x0=par()[["usr"]][1]+1000, x1=par()[["usr"]][1]+2000, y0=par()[["usr"]][3]+500, y1=par()[["usr"]][3]+500, code=3, angle=90, length=0.05)

text(x=par()[["usr"]][1]+1500, y=par()[["usr"]][3]+650, labels="1 km")

#Adding the location of the camera-traps that recorded other communities during that month

# Camera x y location_MCP location_BRB

# CR 554610 9765030 out out

# Lara 552284 9771555 core periphery

# Traverse 554613 9769756 core core

points(x=554610, y=9765030, pch=1, cex=1.2, lwd=2)#CR

points(x=552284, y=9771555, pch=2, cex=1.2, lwd=2)#Lara

points(x=554613, y=9769756, pch=2, cex=1.2, lwd=2)#Traverse

##Adding the legend

#Note that for the supplementary material, we run the legend independently of the map and them put them together as convinient in PowePoint

box.el=150

cols=c("mediumaquamarine", "slategray4", "slategray1",

adjustcolor(col="yellow", alpha.f=0.4), grey(level=0.4, alpha=0.4), adjustcolor(col="lightsteelblue2", alpha.f=0.4), NA, NA,

"black","black", "black")

borders=rep("black", length(cols))

texts=c(paste(c(100, 95, 75), "% MCP", sep=""), "Savannah & Beach", "Swamp", "Sea", "Forest", "95% BRB","75% BRB",

"Foreign camera-trap event (core)","Foreign camera-trap event (non-core)", "Camp")

ltys=c(rep(NA, 7), 1, 2)

for(i in 1:length(texts)){

if(i<=7){

rect(xleft=par()[["usr"]][1]+0.5*box.el, xright=par()[["usr"]][1]+1.5*box.el,

ytop= par()[["usr"]][4]-box.el*0.5*i-box.el*(i-1),

ybottom=par()[["usr"]][4]-box.el*0.5*i-box.el*(i-1)-box.el,

col=cols[i], border="black")

}else if(i<=9){

segments(x0=par()[["usr"]][1]+0.5*box.el, x1=par()[["usr"]][1]+1.5*box.el,

y0=par()[["usr"]][4]-box.el*0.5*i-box.el*(i-1)-box.el/2, y1=par()[["usr"]][4]-box.el*0.5*i-box.el*(i-1)-box.el/2, lty=ltys [i])

}else if(i<=10){

points(x=par()[["usr"]][1]+box.el, y=par()[["usr"]][4]-box.el*0.5*i-box.el*(i-1)-box.el/2, pch=2)

}else if(i<=11){

points(x=par()[["usr"]][1]+box.el, y=par()[["usr"]][4]-box.el*0.5*i-box.el*(i-1)-box.el/2, pch=1)

}else if(i<=12){

points(x=par()[["usr"]][1]+box.el, y=par()[["usr"]][4]-box.el*0.5*i-box.el*(i-1)-box.el/2, pch=8)

}

text(x=par()[["usr"]][1]+1.5*box.el+50, y=par()[["usr"]][4]-box.el*0.5*i-box.el*(i-1)-box.el/2, labels=texts[i], adj=0, cex=0.5)

}

##Adding the title of the map

text(x=par()[["usr"]][1]+7500, y=par()[["usr"]][3]+650, labels="June 2017", adj=0, cex=1.5)

#Saving the map

savePlot(file="C:/Users/Laura/Dropbox/Research/LoangoChimpanzeeProject/201909_intercommunity_study/202006_rekambo_intercommunity_study/202006_temporal_partitioning_maps/202009_intercommunity_temporal_partitioning_201706.png", type="png")

dev.copy2pdf(file="C:/Users/Laura/Dropbox/Research/LoangoChimpanzeeProject/201909_intercommunity_study/202006_rekambo_intercommunity_study/202006_temporal_partitioning_maps/202009_intercommunity_temporal_partitioning_201706.pdf")

####

### # 5.2 Looking at the sightings of other communities relative to the monthly home range of the Rekambo community in August 2017

####

#Preparing the data to calculate the Home Range of the Rekambo community in August 2017

tracks201708<- tracks.2017[tracks.2017$month=="8",]

utm.coords.201708=WGS.to.UTM(long=tracks201708$lon, lat=tracks201708$lat, utm.zone=utm.zone(mean(tracks201708$lon), mean(tracks201708$lat)), avoid.negatives=T, return.zone=F)

tracks.201708=utm.coords.201708

xy201708=SpatialPoints(coords=tracks.201708[, c("long", "lat")])

#Counting amount of data used in the calculations

tracks201708$burst<-as.factor(tracks201708$burst)

str(tracks201708)#burst=tracklog=28; relocations=25 638

##Calculating MCP

to.get=c(100, 95, 75)

MCP201708=vector("list", length(to.get))

names(MCP201708)=to.get

for(i in 1:length(to.get)){

xx201708=mcp(xy201708, percent=to.get[i])

MCP201708[[i]]=xx201708@polygons[[1]]@Polygons[[1]]@coords

}

MCP201708.areas=unlist(lapply(MCP201708, areapl))

MCP201708.areas=MCP201708.areas/(1000^2)

MCP201708.areas

#BRB

#Date for ltraj needs to be in POSIXct format, so we have to transform it

date.brb.201708 <- as.POSIXct(strptime(tracks201708$date,format="%d-%m-%Y %H:%M:%S"))

#Create ltraj object

ltraj.201708=as.ltraj(xy = tracks.201708, date = date.brb.201708, id=F)

#Creating BRB of August 2018

rek.brb.201708 <- BRB(ltraj.201708, D = 1.6, Tmax = 120*60, Lmin = 50, hmin=100,type="UD", grid = 100, extent=1)

#Mapping

par(mar=rep(0.2, 4))

plot(as.matrix(MCP201708[["100"]]), asp=1, xlab="", ylab="", axes=T, type="n", xaxt="n", yaxt="n",

xlim=c(548350, 557873),ylim=c(9765030, 9774195), bty="n")

cols=c("mediumaquamarine", "slategray4", "slategray3", "slategray1")

for(i in 1:length(to.get)){

polygon(MCP201708[[i]], border=NA, col=cols[i])

}

for(i in 1:length(savannah)){

polygon(savannah[[i]], border=NA, col=adjustcolor(col="yellow", alpha.f=0.4))

}

polygon(swamp[[10]], border=NA, col=grey(level=0.4, alpha=0.4))

polygon(sea[[1]], border=NA, col=adjustcolor(col="lightsteelblue2", alpha.f=0.4))

#add brb:

brb201708.cont=rev(c(75, 95))

brb201708.areas=rep(0, length(brb201708.cont))

names(brb201708.areas)=brb201708.cont

for(k in 1:length(brb201708.cont)){

ver.brb201708=getverticeshr(rek.brb.201708, percent=brb201708.cont[k])

for(i in 1:length(ver.brb201708@polygons[[1]]@Polygons)){

polygon(ver.brb201708@polygons[[1]]@Polygons[[i]]@coords, border=grey(level=0.25), col=NA, lty=k)

brb201708.areas[k]=brb201708.areas[k]+areapl(ver.brb201708@polygons[[1]]@Polygons[[i]]@coords)/(1000^2)

}

}

brb201708.areas

#Adding the location of the Ozouga Research Camp

camp.loc=c(x=553601, y=9765888)

points(x=camp.loc["x"], y=camp.loc["y"], pch=8, cex=1.2, lwd=1)

#Adding the scale

arrows(x0=par()[["usr"]][1]+1000, x1=par()[["usr"]][1]+2000, y0=par()[["usr"]][3]+500, y1=par()[["usr"]][3]+500, code=3, angle=90, length=0.05)

text(x=par()[["usr"]][1]+1500, y=par()[["usr"]][3]+650, labels="1 km")

#Adding the location of the camera-traps that recorded other communities during that month

# Camera x y location_MCP location_BRB

# Marc 555861 9767977 periphery out

# Monie 554370 9767606 core core

points(x=555861, y=9767977, pch=1, cex=1.2, lwd=2)#Marc

points(x=554370, y=9767606, pch=2, cex=1.2, lwd=2)#Monie

##Adding the legend

#Note that for the supplementary material, we run the legend independently of the map and them put them together as convinient in PowePoint

box.el=150

cols=c("mediumaquamarine", "slategray4", "slategray1",

adjustcolor(col="yellow", alpha.f=0.4), grey(level=0.4, alpha=0.4), adjustcolor(col="lightsteelblue2", alpha.f=0.4), NA, NA,

"black","black", "black")

borders=rep("black", length(cols))

texts=c(paste(c(100, 95, 75), "% MCP", sep=""), "Savannah & Beach", "Swamp", "Sea", "Forest", "95% BRB","75% BRB",

"Foreign camera-trap event (core)","Foreign camera-trap event (non-core)", "Camp")

ltys=c(rep(NA, 7), 1, 2)

for(i in 1:length(texts)){

if(i<=7){

rect(xleft=par()[["usr"]][1]+0.5*box.el, xright=par()[["usr"]][1]+1.5*box.el,

ytop= par()[["usr"]][4]-box.el*0.5*i-box.el*(i-1),

ybottom=par()[["usr"]][4]-box.el*0.5*i-box.el*(i-1)-box.el,

col=cols[i], border="black")

}else if(i<=9){

segments(x0=par()[["usr"]][1]+0.5*box.el, x1=par()[["usr"]][1]+1.5*box.el,

y0=par()[["usr"]][4]-box.el*0.5*i-box.el*(i-1)-box.el/2, y1=par()[["usr"]][4]-box.el*0.5*i-box.el*(i-1)-box.el/2, lty=ltys [i])

}else if(i<=10){

points(x=par()[["usr"]][1]+box.el, y=par()[["usr"]][4]-box.el*0.5*i-box.el*(i-1)-box.el/2, pch=2)

}else if(i<=11){

points(x=par()[["usr"]][1]+box.el, y=par()[["usr"]][4]-box.el*0.5*i-box.el*(i-1)-box.el/2, pch=1)

}else if(i<=12){

points(x=par()[["usr"]][1]+box.el, y=par()[["usr"]][4]-box.el*0.5*i-box.el*(i-1)-box.el/2, pch=8)

}

text(x=par()[["usr"]][1]+1.5*box.el+50, y=par()[["usr"]][4]-box.el*0.5*i-box.el*(i-1)-box.el/2, labels=texts[i], adj=0, cex=0.5)

}

##Adding the title of the map

text(x=par()[["usr"]][1]+7500, y=par()[["usr"]][3]+650, labels="August 2017", adj=0, cex=1.5)

# Saving the map

savePlot(file="C:/Users/Laura/Dropbox/Research/LoangoChimpanzeeProject/201909_intercommunity_study/202006_rekambo_intercommunity_study/202006_temporal_partitioning_maps/202009_intercommunity_temporal_partitioning_201708.png", type="png")

dev.copy2pdf(file="C:/Users/Laura/Dropbox/Research/LoangoChimpanzeeProject/201909_intercommunity_study/202006_rekambo_intercommunity_study/202006_temporal_partitioning_maps/202009_intercommunity_temporal_partitioning_201708.pdf")

####

### # 5.3 Looking at the sightings of other communities relative to the monthly home range of the Rekambo community in November 2017

####

#Preparing the data to calculate the Home Range of the Rekambo community in November 2017

tracks201711<- tracks.2017[tracks.2017$month=="11",]

utm.coords.201711=WGS.to.UTM(long=tracks201711$lon, lat=tracks201711$lat, utm.zone=utm.zone(mean(tracks201711$lon), mean(tracks201711$lat)), avoid.negatives=T, return.zone=F)

tracks.201711=utm.coords.201711

xy201711=SpatialPoints(coords=tracks.201711[, c("long", "lat")])

#Counting amount of data used in the calculations

tracks201711$burst<-as.factor(tracks201711$burst)

str(tracks201711)#burst=tracklog=28; relocations=25 638

##Calculating MCP

to.get=c(100, 95, 75)

MCP201711=vector("list", length(to.get))

names(MCP201711)=to.get

for(i in 1:length(to.get)){

xx201711=mcp(xy201711, percent=to.get[i])

MCP201711[[i]]=xx201711@polygons[[1]]@Polygons[[1]]@coords

}

MCP201711.areas=unlist(lapply(MCP201711, areapl))

MCP201711.areas=MCP201711.areas/(1000^2)

MCP201711.areas

#BRB

#Date for ltraj needs to be in POSIXct format, so we have to transform it

date.brb.201711 <- as.POSIXct(strptime(tracks201711$date,format="%d-%m-%Y %H:%M:%S"))

#Create ltraj object

ltraj.201711=as.ltraj(xy = tracks.201711, date = date.brb.201711, id=F)

rek.brb.201711 <- BRB(ltraj.201711, D = 1.6, Tmax = 120*60, Lmin = 50, hmin=100,type="UD", grid = 100, extent=1)

#Mapping

par(mar=rep(0.2, 4))

plot(as.matrix(MCP201711[["100"]]), asp=1, xlab="", ylab="", axes=T, type="n", xaxt="n", yaxt="n",

xlim=c(548350, 557873),ylim=c(9765030, 9774195), bty="n")

cols=c("mediumaquamarine", "slategray4", "slategray3", "slategray1")

for(i in 1:length(to.get)){

polygon(MCP201711[[i]], border=NA, col=cols[i])

}

for(i in 1:length(savannah)){

polygon(savannah[[i]], border=NA, col=adjustcolor(col="yellow", alpha.f=0.4))

}

polygon(swamp[[10]], border=NA, col=grey(level=0.4, alpha=0.4))

polygon(sea[[1]], border=NA, col=adjustcolor(col="lightsteelblue2", alpha.f=0.4))

#adding brb:

brb201711.cont=rev(c(75, 95))

brb201711.areas=rep(0, length(brb201711.cont))

names(brb201711.areas)=brb201711.cont

for(k in 1:length(brb201711.cont)){

ver.brb201711=getverticeshr(rek.brb.201711, percent=brb201711.cont[k])

for(i in 1:length(ver.brb201711@polygons[[1]]@Polygons)){

polygon(ver.brb201711@polygons[[1]]@Polygons[[i]]@coords, border=grey(level=0.25), col=NA, lty=k)

brb201711.areas[k]=brb201711.areas[k]+areapl(ver.brb201711@polygons[[1]]@Polygons[[i]]@coords)/(1000^2)

}

}

brb201711.areas

#Adding the location of the Ozouga Research Camp

camp.loc=c(x=553601, y=9765888)

points(x=camp.loc["x"], y=camp.loc["y"], pch=8, cex=1.2, lwd=1)

#Adding the scale

arrows(x0=par()[["usr"]][1]+1000, x1=par()[["usr"]][1]+2000, y0=par()[["usr"]][3]+500, y1=par()[["usr"]][3]+500, code=3, angle=90, length=0.05)

text(x=par()[["usr"]][1]+1500, y=par()[["usr"]][3]+650, labels="1 km")

#Adding the location of the camera-traps that recorded other communities during that month

# Camera x y location_MCP location_BRB

#Traverse 554613 9769756 core core

points(x=554613, y=9769756, pch=2, cex=1.2, lwd=2)#Traverse

##Adding the legend

#Note that for the supplementary material, we run the legend independently of the map and them put them together as convinient in PowePoint

box.el=150

cols=c("mediumaquamarine", "slategray4", "slategray1",

adjustcolor(col="yellow", alpha.f=0.4), grey(level=0.4, alpha=0.4), adjustcolor(col="lightsteelblue2", alpha.f=0.4), NA, NA,

"black","black", "black")

borders=rep("black", length(cols))

texts=c(paste(c(100, 95, 75), "% MCP", sep=""), "Savannah & Beach", "Swamp", "Sea", "Forest", "95% BRB","75% BRB",

"Foreign camera-trap event (core)","Foreign camera-trap event (non-core)", "Camp")

ltys=c(rep(NA, 7), 1, 2)

for(i in 1:length(texts)){

if(i<=7){

rect(xleft=par()[["usr"]][1]+0.5*box.el, xright=par()[["usr"]][1]+1.5*box.el,

ytop= par()[["usr"]][4]-box.el*0.5*i-box.el*(i-1),

ybottom=par()[["usr"]][4]-box.el*0.5*i-box.el*(i-1)-box.el,

col=cols[i], border="black")

}else if(i<=9){

segments(x0=par()[["usr"]][1]+0.5*box.el, x1=par()[["usr"]][1]+1.5*box.el,

y0=par()[["usr"]][4]-box.el*0.5*i-box.el*(i-1)-box.el/2, y1=par()[["usr"]][4]-box.el*0.5*i-box.el*(i-1)-box.el/2, lty=ltys [i])

}else if(i<=10){

points(x=par()[["usr"]][1]+box.el, y=par()[["usr"]][4]-box.el*0.5*i-box.el*(i-1)-box.el/2, pch=2)

}else if(i<=11){

points(x=par()[["usr"]][1]+box.el, y=par()[["usr"]][4]-box.el*0.5*i-box.el*(i-1)-box.el/2, pch=1)

}else if(i<=12){

points(x=par()[["usr"]][1]+box.el, y=par()[["usr"]][4]-box.el*0.5*i-box.el*(i-1)-box.el/2, pch=8)

}

text(x=par()[["usr"]][1]+1.5*box.el+50, y=par()[["usr"]][4]-box.el*0.5*i-box.el*(i-1)-box.el/2, labels=texts[i], adj=0, cex=0.5)

}

##Adding the title of the map

text(x=par()[["usr"]][1]+7500, y=par()[["usr"]][3]+650, labels="November 2017", adj=0, cex=1.5)

#Saving the map

savePlot(file="C:/Users/Laura/Dropbox/Research/LoangoChimpanzeeProject/201909_intercommunity_study/202006_rekambo_intercommunity_study/202006_temporal_partitioning_maps/202009_intercommunity_temporal_partitioning_201711.png", type="png")

dev.copy2pdf(file="C:/Users/Laura/Dropbox/Research/LoangoChimpanzeeProject/201909_intercommunity_study/202006_rekambo_intercommunity_study/202006_temporal_partitioning_maps/202009_intercommunity_temporal_partitioning_201711.pdf")

####

### # 5.4 Looking at the sightings of other communities relative to the monthly home range of the Rekambo community in May 2018

####

#Preparing the data to calculate the Home Range of the Rekambo community in May 2018

tracks201805<- tracks.2018[tracks.2018$month=="6",]

utm.coords.201805=WGS.to.UTM(long=tracks201805$lon, lat=tracks201805$lat, utm.zone=utm.zone(mean(tracks201805$lon), mean(tracks201805$lat)), avoid.negatives=T, return.zone=F)

tracks.201805=utm.coords.201805

xy201805=SpatialPoints(coords=tracks.201805[, c("long", "lat")])

#Counting amount of data used in the calculations

tracks201805$burst<-as.factor(tracks201805$burst)

str(tracks201805)#burst=tracklog=28; relocations=25 638

##Calculating MCP

to.get=c(100, 95, 75)

MCP201805=vector("list", length(to.get))

names(MCP201805)=to.get

for(i in 1:length(to.get)){

xx201805=mcp(xy201805, percent=to.get[i])

MCP201805[[i]]=xx201805@polygons[[1]]@Polygons[[1]]@coords

}

MCP201805.areas=unlist(lapply(MCP201805, areapl))

MCP201805.areas=MCP201805.areas/(1000^2)

MCP201805.areas

#BRB

#Date for ltraj needs to be in POSIXct format, so we have to transform it

date.brb.201805 <- as.POSIXct(strptime(tracks201805$date,format="%d-%m-%Y %H:%M:%S"))

#Create ltraj object

ltraj.201805=as.ltraj(xy = tracks.201805, date = date.brb.201805, id=F)

#Creating the BRB

rek.brb.201805 <- BRB(ltraj.201805, D = 1.6, Tmax = 120*60, Lmin = 50, hmin=100,type="UD", grid = 100, extent=1)

#Mapping

par(mar=rep(0.2, 4))

plot(as.matrix(MCP201805[["100"]]), asp=1, xlab="", ylab="", axes=T, type="n", xaxt="n", yaxt="n",

xlim=c(548350, 557873),ylim=c(9765030, 9774195), bty="n")

cols=c("mediumaquamarine", "slategray4", "slategray3", "slategray1")

for(i in 1:length(to.get)){

polygon(MCP201805[[i]], border=NA, col=cols[i])

}

for(i in 1:length(savannah)){

polygon(savannah[[i]], border=NA, col=adjustcolor(col="yellow", alpha.f=0.4))

}

polygon(swamp[[10]], border=NA, col=grey(level=0.4, alpha=0.4))

polygon(sea[[1]], border=NA, col=adjustcolor(col="lightsteelblue2", alpha.f=0.4))

#adding brb:

brb201805.cont=rev(c(75, 95))

brb201805.areas=rep(0, length(brb201805.cont))

names(brb201805.areas)=brb201805.cont

for(k in 1:length(brb201805.cont)){

ver.brb201805=getverticeshr(rek.brb.201805, percent=brb201805.cont[k])

for(i in 1:length(ver.brb201805@polygons[[1]]@Polygons)){

polygon(ver.brb201805@polygons[[1]]@Polygons[[i]]@coords, border=grey(level=0.25), col=NA, lty=k)

brb201805.areas[k]=brb201805.areas[k]+areapl(ver.brb201805@polygons[[1]]@Polygons[[i]]@coords)/(1000^2)

}

}

brb201805.areas

#Adding the location of the Ozouga Research Camp

camp.loc=c(x=553601, y=9765888)

points(x=camp.loc["x"], y=camp.loc["y"], pch=8, cex=1.2, lwd=1)

#Adding the scale

arrows(x0=par()[["usr"]][1]+1000, x1=par()[["usr"]][1]+2000, y0=par()[["usr"]][3]+500, y1=par()[["usr"]][3]+500, code=3, angle=90, length=0.05)

text(x=par()[["usr"]][1]+1500, y=par()[["usr"]][3]+650, labels="1 km")

#Adding the location of the camera-traps that recorded other communities during that month

# Camera x y location_MCP location_BRB

#Dacryodes 552580 9770907 core core

#golden cat 554346 9771089 core periphery

points(x=552580, y=9770907, pch=2, cex=1.2, lwd=2)#Dacryodes

points(x=554346, y=9771089, pch=2, cex=1.2, lwd=2)#Golden cat

##Adding the legend

#Note that for the supplementary material, we run the legend independently of the map and them put them together as convinient in PowePoint

box.el=150

cols=c("mediumaquamarine", "slategray4", "slategray1",

adjustcolor(col="yellow", alpha.f=0.4), grey(level=0.4, alpha=0.4), adjustcolor(col="lightsteelblue2", alpha.f=0.4), NA, NA,

"black","black", "black")

borders=rep("black", length(cols))

texts=c(paste(c(100, 95, 75), "% MCP", sep=""), "Savannah & Beach", "Swamp", "Sea", "Forest", "95% BRB","75% BRB",

"Foreign camera-trap event (core)","Foreign camera-trap event (non-core)", "Camp")

ltys=c(rep(NA, 7), 1, 2)

for(i in 1:length(texts)){

if(i<=7){

rect(xleft=par()[["usr"]][1]+0.5*box.el, xright=par()[["usr"]][1]+1.5*box.el,

ytop= par()[["usr"]][4]-box.el*0.5*i-box.el*(i-1),

ybottom=par()[["usr"]][4]-box.el*0.5*i-box.el*(i-1)-box.el,

col=cols[i], border="black")

}else if(i<=9){

segments(x0=par()[["usr"]][1]+0.5*box.el, x1=par()[["usr"]][1]+1.5*box.el,

y0=par()[["usr"]][4]-box.el*0.5*i-box.el*(i-1)-box.el/2, y1=par()[["usr"]][4]-box.el*0.5*i-box.el*(i-1)-box.el/2, lty=ltys [i])

}else if(i<=10){

points(x=par()[["usr"]][1]+box.el, y=par()[["usr"]][4]-box.el*0.5*i-box.el*(i-1)-box.el/2, pch=2)

}else if(i<=11){

points(x=par()[["usr"]][1]+box.el, y=par()[["usr"]][4]-box.el*0.5*i-box.el*(i-1)-box.el/2, pch=1)

}else if(i<=12){

points(x=par()[["usr"]][1]+box.el, y=par()[["usr"]][4]-box.el*0.5*i-box.el*(i-1)-box.el/2, pch=8)

}

text(x=par()[["usr"]][1]+1.5*box.el+50, y=par()[["usr"]][4]-box.el*0.5*i-box.el*(i-1)-box.el/2, labels=texts[i], adj=0, cex=0.5)

}

##Adding the title of the map

text(x=par()[["usr"]][1]+7500, y=par()[["usr"]][3]+650, labels="May 2018", adj=0, cex=1.5)

# Saving the map

savePlot(file="C:/Users/Laura/Dropbox/Research/LoangoChimpanzeeProject/201909_intercommunity_study/202006_rekambo_intercommunity_study/202006_temporal_partitioning_maps/202009_intercommunity_temporal_partitioning_201805.png", type="png")

dev.copy2pdf(file="C:/Users/Laura/Dropbox/Research/LoangoChimpanzeeProject/201909_intercommunity_study/202006_rekambo_intercommunity_study/202006_temporal_partitioning_maps/202009_intercommunity_temporal_partitioning_201805.pdf")

####

### # 5.5 Looking at the sightings of other communities relative to the monthly home range of the Rekambo community in June 2018

####

#Preparing the data to calculate the Home Range of the Rekambo community in June 2018

tracks201806<- tracks.2018[tracks.2018$month=="5",]

utm.coords.201806=WGS.to.UTM(long=tracks201806$lon, lat=tracks201806$lat, utm.zone=utm.zone(mean(tracks201806$lon), mean(tracks201806$lat)), avoid.negatives=T, return.zone=F)

tracks.201806=utm.coords.201806

xy201806=SpatialPoints(coords=tracks.201806[, c("long", "lat")])

#Counting amount of data used in the calculations

tracks201806$burst<-as.factor(tracks201806$burst)

str(tracks201806)#burst=tracklog=28; relocations=25 638

##Calculating MCP

to.get=c(100, 95, 75)

MCP201806=vector("list", length(to.get))

names(MCP201806)=to.get

for(i in 1:length(to.get)){

xx201806=mcp(xy201806, percent=to.get[i])

MCP201806[[i]]=xx201806@polygons[[1]]@Polygons[[1]]@coords

}

MCP201806.areas=unlist(lapply(MCP201806, areapl))

MCP201806.areas=MCP201806.areas/(1000^2)

MCP201806.areas

#BRB

#Date for ltraj needs to be in POSIXct format, so we have to transform it

date.brb.201806 <- as.POSIXct(strptime(tracks201806$date,format="%d-%m-%Y %H:%M:%S"))

#Creating ltraj object

ltraj.201806=as.ltraj(xy = tracks.201806, date = date.brb.201806, id=F)

#Creating BRB

rek.brb.201806 <- BRB(ltraj.201806, D = 1.6, Tmax = 120*60, Lmin = 50, hmin=100,type="UD", grid = 100, extent=1)

#Mapping

par(mar=rep(0.2, 4))

plot(as.matrix(MCP201806[["100"]]), asp=1, xlab="", ylab="", axes=T, type="n", xaxt="n", yaxt="n",

xlim=c(548350, 557873),ylim=c(9765030, 9774195), bty="n")

cols=c("mediumaquamarine", "slategray4", "slategray3", "slategray1")

for(i in 1:length(to.get)){

polygon(MCP201806[[i]], border=NA, col=cols[i])

}

for(i in 1:length(savannah)){

polygon(savannah[[i]], border=NA, col=adjustcolor(col="yellow", alpha.f=0.4))

}

polygon(swamp[[10]], border=NA, col=grey(level=0.4, alpha=0.4))

polygon(sea[[1]], border=NA, col=adjustcolor(col="lightsteelblue2", alpha.f=0.4))

# Adding brb:

brb201806.cont=rev(c(75, 95))

brb201806.areas=rep(0, length(brb201806.cont))

names(brb201806.areas)=brb201806.cont

for(k in 1:length(brb201806.cont)){

ver.brb201806=getverticeshr(rek.brb.201806, percent=brb201806.cont[k])

for(i in 1:length(ver.brb201806@polygons[[1]]@Polygons)){

polygon(ver.brb201806@polygons[[1]]@Polygons[[i]]@coords, border=grey(level=0.25), col=NA, lty=k)

brb201806.areas[k]=brb201806.areas[k]+areapl(ver.brb201806@polygons[[1]]@Polygons[[i]]@coords)/(1000^2)

}

}

brb201806.areas

#Adding the location of the Ozouga Research Camp

camp.loc=c(x=553601, y=9765888)

points(x=camp.loc["x"], y=camp.loc["y"], pch=8, cex=1.2, lwd=1)

#Adding the scale

arrows(x0=par()[["usr"]][1]+1000, x1=par()[["usr"]][1]+2000, y0=par()[["usr"]][3]+500, y1=par()[["usr"]][3]+500, code=3, angle=90, length=0.05)

text(x=par()[["usr"]][1]+1500, y=par()[["usr"]][3]+650, labels="1 km")

#Adding the location of the camera-traps that recorded other communities during that month

# Camera x y location_MCP location_BRB

# Lara 552284 9771555 core periphery

# Marcy 554373 9766700 periphery out

points(x=554373, y=9766700, pch=1, cex=1.2, lwd=1)#Marcy

points(x=552284, y=9771555, pch=2, cex=1.2, lwd=2)#Lara

##Adding the legend

#Note that for the supplementary material, we run the legend independently of the map and them put them together as convinient in PowePoint

box.el=150

cols=c("mediumaquamarine", "slategray4", "slategray1",

adjustcolor(col="yellow", alpha.f=0.4), grey(level=0.4, alpha=0.4), adjustcolor(col="lightsteelblue2", alpha.f=0.4), NA, NA,

"black","black", "black")

borders=rep("black", length(cols))

texts=c(paste(c(100, 95, 75), "% MCP", sep=""), "Savannah & Beach", "Swamp", "Sea", "Forest", "95% BRB","75% BRB",

"Foreign camera-trap event (core)","Foreign camera-trap event (non-core)", "Camp")

ltys=c(rep(NA, 7), 1, 2)

for(i in 1:length(texts)){

if(i<=7){

rect(xleft=par()[["usr"]][1]+0.5*box.el, xright=par()[["usr"]][1]+1.5*box.el,

ytop= par()[["usr"]][4]-box.el*0.5*i-box.el*(i-1),

ybottom=par()[["usr"]][4]-box.el*0.5*i-box.el*(i-1)-box.el,

col=cols[i], border="black")

}else if(i<=9){

segments(x0=par()[["usr"]][1]+0.5*box.el, x1=par()[["usr"]][1]+1.5*box.el,

y0=par()[["usr"]][4]-box.el*0.5*i-box.el*(i-1)-box.el/2, y1=par()[["usr"]][4]-box.el*0.5*i-box.el*(i-1)-box.el/2, lty=ltys [i])

}else if(i<=10){

points(x=par()[["usr"]][1]+box.el, y=par()[["usr"]][4]-box.el*0.5*i-box.el*(i-1)-box.el/2, pch=2)

}else if(i<=11){

points(x=par()[["usr"]][1]+box.el, y=par()[["usr"]][4]-box.el*0.5*i-box.el*(i-1)-box.el/2, pch=1)

}else if(i<=12){

points(x=par()[["usr"]][1]+box.el, y=par()[["usr"]][4]-box.el*0.5*i-box.el*(i-1)-box.el/2, pch=8)

}

text(x=par()[["usr"]][1]+1.5*box.el+50, y=par()[["usr"]][4]-box.el*0.5*i-box.el*(i-1)-box.el/2, labels=texts[i], adj=0, cex=0.5)

}

##Adding the title of the map

text(x=par()[["usr"]][1]+7500, y=par()[["usr"]][3]+650, labels="June 2018", adj=0, cex=1.5)

# Saving the map

savePlot(file="C:/Users/Laura/Dropbox/Research/LoangoChimpanzeeProject/201909_intercommunity_study/202006_rekambo_intercommunity_study/202006_temporal_partitioning_maps/202009_intercommunity_temporal_partitioning_201806.png", type="png")

dev.copy2pdf(file="C:/Users/Laura/Dropbox/Research/LoangoChimpanzeeProject/201909_intercommunity_study/202006_rekambo_intercommunity_study/202006_temporal_partitioning_maps/202009_intercommunity_temporal_partitioning_201806.pdf")

####

### # 5.6 Looking at the sightings of other communities relative to the monthly home range of the Rekambo community in July 2018

####

#Preparing the data to calculate the Home Range of the Rekambo community in July 2018

tracks201807<- tracks.2018[tracks.2018$month=="7",]

utm.coords.201807=WGS.to.UTM(long=tracks201807$lon, lat=tracks201807$lat, utm.zone=utm.zone(mean(tracks201807$lon), mean(tracks201807$lat)), avoid.negatives=T, return.zone=F)

tracks.201807=utm.coords.201807

xy201807=SpatialPoints(coords=tracks.201807[, c("long", "lat")])

#Counting amount of data used in the calculations

tracks201807$burst<-as.factor(tracks201807$burst)

str(tracks201807)#burst=tracklog=28; relocations=25 638

##Calculating MCP

to.get=c(100, 95, 75)

MCP201807=vector("list", length(to.get))

names(MCP201807)=to.get

for(i in 1:length(to.get)){

xx201807=mcp(xy201807, percent=to.get[i])

MCP201807[[i]]=xx201807@polygons[[1]]@Polygons[[1]]@coords

}

MCP201807.areas=unlist(lapply(MCP201807, areapl))

MCP201807.areas=MCP201807.areas/(1000^2)

MCP201807.areas

#BRB

# Date for ltraj needs to be in POSIXct format, so we have to transform it

date.brb.201807 <- as.POSIXct(strptime(tracks201807$date,format="%d-%m-%Y %H:%M:%S"))

# Create ltraj object

ltraj.201807=as.ltraj(xy = tracks.201807, date = date.brb.201807, id=F)

# Creating BRB

rek.brb.201807 <- BRB(ltraj.201807, D = 1.6, Tmax = 120*60, Lmin = 50, hmin=100,type="UD", grid = 100, extent=1)

#Mapping

par(mar=rep(0.2, 4))

plot(as.matrix(MCP201807[["100"]]), asp=1, xlab="", ylab="", axes=T, type="n", xaxt="n", yaxt="n",

xlim=c(548350, 557873),ylim=c(9765030, 9774195), bty="n")

cols=c("mediumaquamarine", "slategray4", "slategray3", "slategray1")

for(i in 1:length(to.get)){

polygon(MCP201807[[i]], border=NA, col=cols[i])

}

for(i in 1:length(savannah)){

polygon(savannah[[i]], border=NA, col=adjustcolor(col="yellow", alpha.f=0.4))

}

polygon(swamp[[10]], border=NA, col=grey(level=0.4, alpha=0.4))

polygon(sea[[1]], border=NA, col=adjustcolor(col="lightsteelblue2", alpha.f=0.4))

#adding brb:

brb201807.cont=rev(c(75, 95))

brb201807.areas=rep(0, length(brb201807.cont))

names(brb201807.areas)=brb201807.cont

for(k in 1:length(brb201807.cont)){

ver.brb201807=getverticeshr(rek.brb.201807, percent=brb201807.cont[k])

for(i in 1:length(ver.brb201807@polygons[[1]]@Polygons)){

polygon(ver.brb201807@polygons[[1]]@Polygons[[i]]@coords, border=grey(level=0.25), col=NA, lty=k)

brb201807.areas[k]=brb201807.areas[k]+areapl(ver.brb201807@polygons[[1]]@Polygons[[i]]@coords)/(1000^2)

}

}

brb201807.areas

#Adding the location of the Ozouga Research Camp

camp.loc=c(x=553601, y=9765888)

points(x=camp.loc["x"], y=camp.loc["y"], pch=8, cex=1.2, lwd=1)

#Adding the scale

arrows(x0=par()[["usr"]][1]+1000, x1=par()[["usr"]][1]+2000, y0=par()[["usr"]][3]+500, y1=par()[["usr"]][3]+500, code=3, angle=90, length=0.05)

text(x=par()[["usr"]][1]+1500, y=par()[["usr"]][3]+650, labels="1 km")

#Adding the location of the camera-traps that recorded other communities during that month

#Monie 554370 9767606 core core

points(x=554370, y=9767606, pch=2, cex=1.2, lwd=2)#Monie

##Adding the legend

#Note that for the supplementary material, we run the legend independently of the map and them put them together as convinient in PowePoint

box.el=150

cols=c("mediumaquamarine", "slategray4", "slategray1",

adjustcolor(col="yellow", alpha.f=0.4), grey(level=0.4, alpha=0.4), adjustcolor(col="lightsteelblue2", alpha.f=0.4), NA, NA,

"black","black", "black")

borders=rep("black", length(cols))

texts=c(paste(c(100, 95, 75), "% MCP", sep=""), "Savannah & Beach", "Swamp", "Sea", "Forest", "95% BRB","75% BRB",

"Foreign camera-trap event (core)","Foreign camera-trap event (non-core)", "Camp")

ltys=c(rep(NA, 7), 1, 2)

for(i in 1:length(texts)){

if(i<=7){

rect(xleft=par()[["usr"]][1]+0.5*box.el, xright=par()[["usr"]][1]+1.5*box.el,

ytop= par()[["usr"]][4]-box.el*0.5*i-box.el*(i-1),

ybottom=par()[["usr"]][4]-box.el*0.5*i-box.el*(i-1)-box.el,

col=cols[i], border="black")

}else if(i<=9){

segments(x0=par()[["usr"]][1]+0.5*box.el, x1=par()[["usr"]][1]+1.5*box.el,

y0=par()[["usr"]][4]-box.el*0.5*i-box.el*(i-1)-box.el/2, y1=par()[["usr"]][4]-box.el*0.5*i-box.el*(i-1)-box.el/2, lty=ltys [i])

}else if(i<=10){

points(x=par()[["usr"]][1]+box.el, y=par()[["usr"]][4]-box.el*0.5*i-box.el*(i-1)-box.el/2, pch=2)

}else if(i<=11){

points(x=par()[["usr"]][1]+box.el, y=par()[["usr"]][4]-box.el*0.5*i-box.el*(i-1)-box.el/2, pch=1)

}else if(i<=12){

points(x=par()[["usr"]][1]+box.el, y=par()[["usr"]][4]-box.el*0.5*i-box.el*(i-1)-box.el/2, pch=8)

}

text(x=par()[["usr"]][1]+1.5*box.el+50, y=par()[["usr"]][4]-box.el*0.5*i-box.el*(i-1)-box.el/2, labels=texts[i], adj=0, cex=0.5)

}

##Adding the title of the map

text(x=par()[["usr"]][1]+7500, y=par()[["usr"]][3]+650, labels="July 2018", adj=0, cex=1.5)

#Saving the map

savePlot(file="C:/Users/Laura/Dropbox/Research/LoangoChimpanzeeProject/201909_intercommunity_study/202006_rekambo_intercommunity_study/202009_temporal_partitioning_maps/202009_intercommunity_temporal_partitioning_201807.png", type="png")

dev.copy2pdf(file="C:/Users/Laura/Dropbox/Research/LoangoChimpanzeeProject/201909_intercommunity_study/202006_rekambo_intercommunity_study/202009_temporal_partitioning_maps/202009_intercommunity_temporal_partitioning_201807.pdf")

####

### # 5.7 Looking at the sightings of other communities relative to the monthly home range of the Rekambo community in September 2018

####

#Preparing the data to calculate the Home Range of the Rekambo community in September 2018

tracks201809<- tracks.2018[tracks.2018$month=="9",]

utm.coords.201809=WGS.to.UTM(long=tracks201809$lon, lat=tracks201809$lat, utm.zone=utm.zone(mean(tracks201809$lon), mean(tracks201809$lat)), avoid.negatives=T, return.zone=F)

tracks.201809=utm.coords.201809

xy201809=SpatialPoints(coords=tracks.201809[, c("long", "lat")])

#Counting amount of data used in the calculations

tracks201809$burst<-as.factor(tracks201809$burst)

str(tracks201809)#burst=tracklog=28; relocations=25 638

##Calculating MCP

to.get=c(100, 95, 75)

MCP201809=vector("list", length(to.get))

names(MCP201809)=to.get

for(i in 1:length(to.get)){

xx201809=mcp(xy201809, percent=to.get[i])

MCP201809[[i]]=xx201809@polygons[[1]]@Polygons[[1]]@coords

}

MCP201809.areas=unlist(lapply(MCP201809, areapl))

MCP201809.areas=MCP201809.areas/(1000^2)

MCP201809.areas

#BRB

#Date for ltraj needs to be in POSIXct format, so we have to transform it

date.brb.201809 <- as.POSIXct(strptime(tracks201809$date,format="%d-%m-%Y %H:%M:%S"))

#Create ltraj object

ltraj.201809=as.ltraj(xy = tracks.201809, date = date.brb.201809, id=F)

#Creating BRB

rek.brb.201809 <- BRB(ltraj.201809, D = 1.6, Tmax = 120*60, Lmin = 50, hmin=100,type="UD", grid = 100, extent=1)

#Mapping

par(mar=rep(0.2, 4))

plot(as.matrix(MCP201809[["100"]]), asp=1, xlab="", ylab="", axes=T, type="n", xaxt="n", yaxt="n",

xlim=c(548350, 557873),ylim=c(9765030, 9774195), bty="n")

cols=c("mediumaquamarine", "slategray4", "slategray3", "slategray1")

for(i in 1:length(to.get)){

polygon(MCP201809[[i]], border=NA, col=cols[i])

}

for(i in 1:length(savannah)){

polygon(savannah[[i]], border=NA, col=adjustcolor(col="yellow", alpha.f=0.4))

}

polygon(swamp[[10]], border=NA, col=grey(level=0.4, alpha=0.4))

polygon(sea[[1]], border=NA, col=adjustcolor(col="lightsteelblue2", alpha.f=0.4))

#adding brb:

brb201809.cont=rev(c(75, 95))

brb201809.areas=rep(0, length(brb201809.cont))

names(brb201809.areas)=brb201809.cont

for(k in 1:length(brb201809.cont)){

ver.brb201809=getverticeshr(rek.brb.201809, percent=brb201809.cont[k])

for(i in 1:length(ver.brb201809@polygons[[1]]@Polygons)){

polygon(ver.brb201809@polygons[[1]]@Polygons[[i]]@coords, border=grey(level=0.25), col=NA, lty=k)

brb201809.areas[k]=brb201809.areas[k]+areapl(ver.brb201809@polygons[[1]]@Polygons[[i]]@coords)/(1000^2)

}

}

brb201809.areas

#Adding the location of the Ozouga Research Camp

camp.loc=c(x=553601, y=9765888)

points(x=camp.loc["x"], y=camp.loc["y"], pch=8, cex=1.2, lwd=1)

#Adding the scale

arrows(x0=par()[["usr"]][1]+1000, x1=par()[["usr"]][1]+2000, y0=par()[["usr"]][3]+500, y1=par()[["usr"]][3]+500, code=3, angle=90, length=0.05)

text(x=par()[["usr"]][1]+1500, y=par()[["usr"]][3]+650, labels="1 km")

#Adding the location of the camera-traps that recorded other communities during that month

# Camera x y location_MCP location_BRB

# CR 554610 9765030 out out

# Lara 552284 9771555 core periphery

#Dacryodes 552580 9770907 core core

# Marcy 554373 9766700 periphery out

# Lulu 553297 9768936 core core

points(x=554610, y=9765030, pch=1, cex=1.2, lwd=2)#CR

points(x=552284, y=9771555, pch=2, cex=1.2, lwd=2)#Lara

points(x=554373, y=9766700, pch=1, cex=1.2, lwd=2)#Marcy

points(x=552580, y=9770907, pch=2, cex=1.2, lwd=2)#Dacryodes

points(x=553297, y=9768936, pch=2, cex=1.2, lwd=2)#Lulu

##Adding the legend

#Note that for the supplementary material, we run the legend independently of the map and them put them together as convinient in PowePoint

box.el=150

cols=c("mediumaquamarine", "slategray4", "slategray1",

adjustcolor(col="yellow", alpha.f=0.4), grey(level=0.4, alpha=0.4), adjustcolor(col="lightsteelblue2", alpha.f=0.4), NA, NA,

"black","black", "black")

borders=rep("black", length(cols))

texts=c(paste(c(100, 95, 75), "% MCP", sep=""), "Savannah & Beach", "Swamp", "Sea", "Forest", "95% BRB","75% BRB",

"Foreign camera-trap event (core)","Foreign camera-trap event (non-core)", "Camp")

ltys=c(rep(NA, 7), 1, 2)

for(i in 1:length(texts)){

if(i<=7){

rect(xleft=par()[["usr"]][1]+0.5*box.el, xright=par()[["usr"]][1]+1.5*box.el,

ytop= par()[["usr"]][4]-box.el*0.5*i-box.el*(i-1),

ybottom=par()[["usr"]][4]-box.el*0.5*i-box.el*(i-1)-box.el,

col=cols[i], border="black")

}else if(i<=9){

segments(x0=par()[["usr"]][1]+0.5*box.el, x1=par()[["usr"]][1]+1.5*box.el,

y0=par()[["usr"]][4]-box.el*0.5*i-box.el*(i-1)-box.el/2, y1=par()[["usr"]][4]-box.el*0.5*i-box.el*(i-1)-box.el/2, lty=ltys [i])

}else if(i<=10){

points(x=par()[["usr"]][1]+box.el, y=par()[["usr"]][4]-box.el*0.5*i-box.el*(i-1)-box.el/2, pch=2)

}else if(i<=11){

points(x=par()[["usr"]][1]+box.el, y=par()[["usr"]][4]-box.el*0.5*i-box.el*(i-1)-box.el/2, pch=1)

}else if(i<=12){

points(x=par()[["usr"]][1]+box.el, y=par()[["usr"]][4]-box.el*0.5*i-box.el*(i-1)-box.el/2, pch=8)

}

text(x=par()[["usr"]][1]+1.5*box.el+50, y=par()[["usr"]][4]-box.el*0.5*i-box.el*(i-1)-box.el/2, labels=texts[i], adj=0, cex=0.5)

}

##Adding the title of the map

text(x=par()[["usr"]][1]+7500, y=par()[["usr"]][3]+650, labels="September 2018", adj=0, cex=1.5)

#Saving the map

savePlot(file="C:/Users/Laura/Dropbox/Research/LoangoChimpanzeeProject/201909_intercommunity_study/202006_rekambo_intercommunity_study/202006_temporal_partitioning_maps/202006_intercommunity_temporal_partitioning_201809_no50.png", type="png")

dev.copy2pdf(file="C:/Users/Laura/Dropbox/Research/LoangoChimpanzeeProject/201909_intercommunity_study/202006_rekambo_intercommunity_study/202006_temporal_partitioning_maps/202006_intercommunity_temporal_partitioning_201809_no50.pdf")

####

### # 5.8 Looking at the sightings of other communities relative to the monthly home range of the Rekambo community in December 2018

####

#Preparing the data to calculate the Home Range of the Rekambo community in December 2018

tracks201812<- tracks.2018[tracks.2018$month=="12",]

utm.coords.201812=WGS.to.UTM(long=tracks201812$lon, lat=tracks201812$lat, utm.zone=utm.zone(mean(tracks201812$lon), mean(tracks201812$lat)), avoid.negatives=T, return.zone=F)

tracks.201812=utm.coords.201812

xy201812=SpatialPoints(coords=tracks.201812[, c("long", "lat")])

#Counting amount of data used in the calculations

tracks201812$burst<-as.factor(tracks201812$burst)

str(tracks201812)#burst=tracklog=28; relocations=25 638

##Calculating MCP

to.get=c(100, 95, 75)

MCP201812=vector("list", length(to.get))

names(MCP201812)=to.get

for(i in 1:length(to.get)){

xx201812=mcp(xy201812, percent=to.get[i])

MCP201812[[i]]=xx201812@polygons[[1]]@Polygons[[1]]@coords

}

MCP201812.areas=unlist(lapply(MCP201812, areapl))

MCP201812.areas=MCP201812.areas/(1000^2)

MCP201812.areas

#BRB

# Date for ltraj needs to be in POSIXct format, so we have to transform it

date.brb.201812 <- as.POSIXct(strptime(tracks201812$date,format="%d-%m-%Y %H:%M:%S"))

# Create ltraj object

ltraj.201812=as.ltraj(xy = tracks.201812, date = date.brb.201812, id=F)

# Creating the BRB

rek.brb.201812 <- BRB(ltraj.201812, D = 1.6, Tmax = 120*60, Lmin = 50, hmin=100,type="UD", grid = 100, extent=1)

#Mapping

par(mar=rep(0.2, 4))

plot(as.matrix(MCP201812[["100"]]), asp=1, xlab="", ylab="", axes=T, type="n", xaxt="n", yaxt="n",

xlim=c(548350, 557873),ylim=c(9765030, 9774195), bty="n")

cols=c("mediumaquamarine", "slategray4", "slategray3", "slategray1")

for(i in 1:length(to.get)){

polygon(MCP201812[[i]], border=NA, col=cols[i])

}

for(i in 1:length(savannah)){

polygon(savannah[[i]], border=NA, col=adjustcolor(col="yellow", alpha.f=0.4))

}

polygon(swamp[[10]], border=NA, col=grey(level=0.4, alpha=0.4))

polygon(sea[[1]], border=NA, col=adjustcolor(col="lightsteelblue2", alpha.f=0.4))

#add brb:

brb201812.cont=rev(c(75, 95))

brb201812.areas=rep(0, length(brb201812.cont))

names(brb201812.areas)=brb201812.cont

for(k in 1:length(brb201812.cont)){

ver.brb201812=getverticeshr(rek.brb.201812, percent=brb201812.cont[k])

for(i in 1:length(ver.brb201812@polygons[[1]]@Polygons)){

polygon(ver.brb201812@polygons[[1]]@Polygons[[i]]@coords, border=grey(level=0.25), col=NA, lty=k)

brb201812.areas[k]=brb201812.areas[k]+areapl(ver.brb201812@polygons[[1]]@Polygons[[i]]@coords)/(1000^2)

}

}

brb201812.areas

#Adding the location of the Ozouga Research Camp

camp.loc=c(x=553601, y=9765888)

points(x=camp.loc["x"], y=camp.loc["y"], pch=8, cex=1.2, lwd=1)

#Adding the scale

arrows(x0=par()[["usr"]][1]+1000, x1=par()[["usr"]][1]+2000, y0=par()[["usr"]][3]+500, y1=par()[["usr"]][3]+500, code=3, angle=90, length=0.05)

text(x=par()[["usr"]][1]+1500, y=par()[["usr"]][3]+650, labels="1 km")

#Adding the location of the camera-traps that recorded other communities during that month

# Camera x y location_MCP location_BRB

#3 sacco 553931 9770445 core core

#Rekambo2 551714 9772153 periphery out

points(x=551714, y=9772153, pch=1, cex=1.2, lwd=2)#Rekambo2

points(x=553931, y=9770445 , pch=2, cex=1.2, lwd=2)#Lara

##Adding the legend

#Note that for the supplementary material, we run the legend independently of the map and them put them together as convinient in PowePoint

box.el=150

cols=c("mediumaquamarine", "slategray4", "slategray1",

adjustcolor(col="yellow", alpha.f=0.4), grey(level=0.4, alpha=0.4), adjustcolor(col="lightsteelblue2", alpha.f=0.4), NA, NA,

"black","black", "black")

borders=rep("black", length(cols))

texts=c(paste(c(100, 95, 75), "% MCP", sep=""), "Savannah & Beach", "Swamp", "Sea", "Forest", "95% BRB","75% BRB",

"Foreign camera-trap event (core)","Foreign camera-trap event (non-core)", "Camp")

ltys=c(rep(NA, 7), 1, 2)

for(i in 1:length(texts)){

if(i<=7){

rect(xleft=par()[["usr"]][1]+0.5*box.el, xright=par()[["usr"]][1]+1.5*box.el,

ytop= par()[["usr"]][4]-box.el*0.5*i-box.el*(i-1),

ybottom=par()[["usr"]][4]-box.el*0.5*i-box.el*(i-1)-box.el,

col=cols[i], border="black")

}else if(i<=9){

segments(x0=par()[["usr"]][1]+0.5*box.el, x1=par()[["usr"]][1]+1.5*box.el,

y0=par()[["usr"]][4]-box.el*0.5*i-box.el*(i-1)-box.el/2, y1=par()[["usr"]][4]-box.el*0.5*i-box.el*(i-1)-box.el/2, lty=ltys [i])

}else if(i<=10){

points(x=par()[["usr"]][1]+box.el, y=par()[["usr"]][4]-box.el*0.5*i-box.el*(i-1)-box.el/2, pch=2)

}else if(i<=11){

points(x=par()[["usr"]][1]+box.el, y=par()[["usr"]][4]-box.el*0.5*i-box.el*(i-1)-box.el/2, pch=1)

}else if(i<=12){

points(x=par()[["usr"]][1]+box.el, y=par()[["usr"]][4]-box.el*0.5*i-box.el*(i-1)-box.el/2, pch=8)

}

text(x=par()[["usr"]][1]+1.5*box.el+50, y=par()[["usr"]][4]-box.el*0.5*i-box.el*(i-1)-box.el/2, labels=texts[i], adj=0, cex=0.5)

}

##Adding the title of the map

text(x=par()[["usr"]][1]+7500, y=par()[["usr"]][3]+650, labels="December 2018", adj=0, cex=1.5)

# Saving the map

savePlot(file="C:/Users/Laura/Dropbox/Research/LoangoChimpanzeeProject/201909_intercommunity_study/202006_rekambo_intercommunity_study/202006_temporal_partitioning_maps/202009_intercommunity_temporal_partitioning_201812.png", type="png")

dev.copy2pdf(file="C:/Users/Laura/Dropbox/Research/LoangoChimpanzeeProject/201909_intercommunity_study/202006_rekambo_intercommunity_study/202006_temporal_partitioning_maps/202009_intercommunity_temporal_partitioning_201812.pdf")

####

### # 5.9 Looking at the sightings of other communities relative to the monthly home range of the Rekambo community in January 2019

####

#Preparing the data to calculate the Home Range of the Rekambo community in January 2019

tracks201901<- tracks.2019[tracks.2019$month=="1",]

utm.coords.201901=WGS.to.UTM(long=tracks201901$lon, lat=tracks201901$lat, utm.zone=utm.zone(mean(tracks201901$lon), mean(tracks201901$lat)), avoid.negatives=T, return.zone=F)

tracks.201901=utm.coords.201901

xy201901=SpatialPoints(coords=tracks.201901[, c("long", "lat")])

#Counting amount of data used in the calculations

tracks201901$burst<-as.factor(tracks201901$burst)

str(tracks201901)#burst=tracklog=28; relocations=25 638

##Calculating MCP

to.get=c(100, 95, 75)

MCP201901=vector("list", length(to.get))

names(MCP201901)=to.get

for(i in 1:length(to.get)){

xx201901=mcp(xy201901, percent=to.get[i])

MCP201901[[i]]=xx201901@polygons[[1]]@Polygons[[1]]@coords

}

MCP201901.areas=unlist(lapply(MCP201901, areapl))

MCP201901.areas=MCP201901.areas/(1000^2)

MCP201901.areas

#BRB

#Date for ltraj needs to be in POSIXct format, so we have to transform it

date.brb.201901 <- as.POSIXct(strptime(tracks201901$date,format="%d-%m-%Y %H:%M:%S"))

#Create ltraj object

ltraj.201901=as.ltraj(xy = tracks.201901, date = date.brb.201901, id=F)

#Creating the BRB

rek.brb.201901 <- BRB(ltraj.201901, D = 1.6, Tmax = 120*60, Lmin = 50, hmin=100,type="UD", grid = 100, extent=1)

#Mapping

par(mar=rep(0.2, 4))

plot(as.matrix(MCP201901[["100"]]), asp=1, xlab="", ylab="", axes=T, type="n", xaxt="n", yaxt="n",

xlim=c(548350, 557873),ylim=c(9765030, 9774195), bty="n")

cols=c("mediumaquamarine", "slategray4", "slategray3", "slategray1")

for(i in 1:length(to.get)){

polygon(MCP201901[[i]], border=NA, col=cols[i])

}

for(i in 1:length(savannah)){

polygon(savannah[[i]], border=NA, col=adjustcolor(col="yellow", alpha.f=0.4))

}

polygon(swamp[[10]], border=NA, col=grey(level=0.4, alpha=0.4))

polygon(sea[[1]], border=NA, col=adjustcolor(col="lightsteelblue2", alpha.f=0.4))

#adding the brb:

brb201901.cont=rev(c(75, 95))

brb201901.areas=rep(0, length(brb201901.cont))

names(brb201901.areas)=brb201901.cont

for(k in 1:length(brb201901.cont)){

ver.brb201901=getverticeshr(rek.brb.201901, percent=brb201901.cont[k])

for(i in 1:length(ver.brb201901@polygons[[1]]@Polygons)){

polygon(ver.brb201901@polygons[[1]]@Polygons[[i]]@coords, border=grey(level=0.25), col=NA, lty=k)

brb201901.areas[k]=brb201901.areas[k]+areapl(ver.brb201901@polygons[[1]]@Polygons[[i]]@coords)/(1000^2)

}

}

brb201901.areas

#Adding the location of the Ozouga Research Camp

camp.loc=c(x=553601, y=9765888)

points(x=camp.loc["x"], y=camp.loc["y"], pch=8, cex=1.2, lwd=1)

#Adding the scale

arrows(x0=par()[["usr"]][1]+1000, x1=par()[["usr"]][1]+2000, y0=par()[["usr"]][3]+500, y1=par()[["usr"]][3]+500, code=3, angle=90, length=0.05)

text(x=par()[["usr"]][1]+1500, y=par()[["usr"]][3]+650, labels="1 km")

#Adding the location of the camera-traps that recorded other communities during that month

# Camera x y location_MCP location_BRB

#Perroquet 554325 9770544 core core

points(x=554325, y=9770544, pch=2, cex=1.2, lwd=2)#Perroquet

##Adding the legend

#Note that for the supplementary material, we run the legend independently of the map and them put them together as convinient in PowePoint

box.el=150

cols=c("mediumaquamarine", "slategray4", "slategray1",

adjustcolor(col="yellow", alpha.f=0.4), grey(level=0.4, alpha=0.4), adjustcolor(col="lightsteelblue2", alpha.f=0.4), NA, NA,

"black","black", "black")

borders=rep("black", length(cols))

texts=c(paste(c(100, 95, 75), "% MCP", sep=""), "Savannah & Beach", "Swamp", "Sea", "Forest", "95% BRB","75% BRB",

"Foreign camera-trap event (core)","Foreign camera-trap event (non-core)", "Camp")

ltys=c(rep(NA, 7), 1, 2)

for(i in 1:length(texts)){

if(i<=7){

rect(xleft=par()[["usr"]][1]+0.5*box.el, xright=par()[["usr"]][1]+1.5*box.el,

ytop= par()[["usr"]][4]-box.el*0.5*i-box.el*(i-1),

ybottom=par()[["usr"]][4]-box.el*0.5*i-box.el*(i-1)-box.el,

col=cols[i], border="black")

}else if(i<=9){

segments(x0=par()[["usr"]][1]+0.5*box.el, x1=par()[["usr"]][1]+1.5*box.el,

y0=par()[["usr"]][4]-box.el*0.5*i-box.el*(i-1)-box.el/2, y1=par()[["usr"]][4]-box.el*0.5*i-box.el*(i-1)-box.el/2, lty=ltys [i])

}else if(i<=10){

points(x=par()[["usr"]][1]+box.el, y=par()[["usr"]][4]-box.el*0.5*i-box.el*(i-1)-box.el/2, pch=2)

}else if(i<=11){

points(x=par()[["usr"]][1]+box.el, y=par()[["usr"]][4]-box.el*0.5*i-box.el*(i-1)-box.el/2, pch=1)

}else if(i<=12){

points(x=par()[["usr"]][1]+box.el, y=par()[["usr"]][4]-box.el*0.5*i-box.el*(i-1)-box.el/2, pch=8)

}

text(x=par()[["usr"]][1]+1.5*box.el+50, y=par()[["usr"]][4]-box.el*0.5*i-box.el*(i-1)-box.el/2, labels=texts[i], adj=0, cex=0.5)

}

##Adding the title of the map

text(x=par()[["usr"]][1]+7500, y=par()[["usr"]][3]+650, labels="January 2019", adj=0, cex=1.5)

#Saving the map

savePlot(file="C:/Users/Laura/Dropbox/Research/LoangoChimpanzeeProject/201909_intercommunity_study/202006_rekambo_intercommunity_study/202006_temporal_partitioning_maps/202009_intercommunity_temporal_partitioning_201901.png", type="png")

dev.copy2pdf(file="C:/Users/Laura/Dropbox/Research/LoangoChimpanzeeProject/201909_intercommunity_study/202006_rekambo_intercommunity_study/202006_temporal_partitioning_maps/202009_intercommunity_temporal_partitioning_201901.pdf")
